# The Impact of a Digital Weight Loss Intervention on Health Care Resource Utilization and Costs Compared Between Users and Nonusers With Overweight and Obesity: Retrospective Analysis Study

**DOI:** 10.2196/47473

**Published:** 2023-08-24

**Authors:** Ellen Siobhan Mitchell, Alexander Fabry, Annabell Suh Ho, Christine N May, Matthew Baldwin, Paige Blanco, Kyle Smith, Andreas Michaelides, Mostafa Shokoohi, Michael West, Kim Gotera, Omnya El Massad, Anna Zhou

**Affiliations:** 1 Academic Research Noom, Inc New York City, NY United States; 2 Eversana Burlington, ON Canada; 3 Eversana Sydney, NS Canada

**Keywords:** mobile health, mHealth, obesity, overweight, Noom Weight, digital weight loss intervention, health care resource utilization, costs, electronic health record, EHR, insurance claims, inverse probability of treatment weighting, IPTW, mobile phone

## Abstract

**Background:**

The Noom Weight program is a smartphone-based weight management program that uses cognitive behavioral therapy techniques to motivate users to achieve weight loss through a comprehensive lifestyle intervention.

**Objective:**

This retrospective database analysis aimed to evaluate the impact of Noom Weight use on health care resource utilization (HRU) and health care costs among individuals with overweight and obesity.

**Methods:**

Electronic health record data, insurance claims data, and Noom Weight program data were used to conduct the analysis. The study included 43,047 Noom Weight users and 14,555 non–Noom Weight users aged between 18 and 80 years with a BMI of ≥25 kg/m² and residing in the United States. The index date was defined as the first day of a 3-month treatment window during which Noom Weight was used at least once per week on average. Inverse probability treatment weighting was used to balance sociodemographic covariates between the 2 cohorts. HRU and costs for inpatient visits, outpatient visits, telehealth visits, surgeries, and prescriptions were analyzed.

**Results:**

Within 12 months after the index date, Noom Weight users had less inpatient costs (mean difference [MD] −US $20.10, 95% CI −US $30.08 to −US $10.12), less outpatient costs (MD −US $124.33, 95% CI −US $159.76 to −US $88.89), less overall prescription costs (MD −US $313.82, 95% CI −US $565.42 to −US $62.21), and less overall health care costs (MD −US $450.39, 95% CI −US $706.28 to −US $194.50) per user than non–Noom Weight users. In terms of HRU, Noom Weight users had fewer inpatient visits (MD −0.03, 95% CI −0.04 to −0.03), fewer outpatient visits (MD −0.78, 95% CI −0.93 to −0.62), fewer surgeries (MD −0.01, 95% CI −0.01 to 0.00), and fewer prescriptions (MD −1.39, 95% CI −1.76 to −1.03) per user than non–Noom Weight users. Among a subset of individuals with 24-month follow-up data, Noom Weight users incurred lower overall prescription costs (MD −US $1139.52, 95% CI −US $1972.21 to −US $306.83) and lower overall health care costs (MD −US $1219.06, 95% CI −US $2061.56 to −US $376.55) per user than non–Noom Weight users. The key differences were associated with reduced prescription use.

**Conclusions:**

Noom Weight use is associated with lower HRU and costs than non–Noom Weight use, with potential cost savings of up to US $1219.06 per user at 24 months after the index date. These findings suggest that Noom Weight could be a cost-effective weight management program for individuals with overweight and obesity. This study provides valuable evidence for health care providers and payers in evaluating the potential benefits of digital weight loss interventions such as Noom Weight.

## Introduction

### Background

Rising rates of obesity globally [1] have led to substantial increases in related health care expenditures. From 2000 to 2018, the age-adjusted rate of obesity in the United States increased from 30.5% to 42.4%, with 76.5% of the adult population classified as either overweight or obese in 2018 [2]. Between 1998 and 2016, obesity-related health care spending in the United States increased from US $111.7 to US $170.3 billion [3] to US $182 to US $288 billion (2021 US dollars) [4,5]. Obesity is associated with an increase in direct annual medical costs ranging from US $1961 [5] to US $3423 [4] per individual (2021 US dollars). Furthermore, obesity-related comorbidities are among the greatest contributors to total US annual medical expenditures, including US $126 billion for diabetes, US $101.2 billion for ischemic heart disease, US $89.5 billion for hypertension, and US $29.9 billion for hyperlipidemia (2021 US dollars) [6].

There is a pressing need for effective strategies to address these rising costs and the growing prevalence of obesity [7]. Standard dietary interventions that maintain an energy deficit typically produce an average maximal weight loss of 4 to 12 kg after 6 months, with smaller sustained losses of 4 to 10 kg after 1 year and only 3 to 4 kg after 2 years [8]. Lifestyle changes are typically required to sustain weight losses, and, as such, a lifestyle intervention is an effective approach [9-11].

In-person interventions, although effective, can be time-consuming, expensive, and thus unappealing to many potential participants [12,13]. In addition to these barriers, limited program availability and potential lack of reimbursement [14,15] further limit widespread participation. Remote interventions using mobile health (mHealth) technologies such as telephone calls, SMS text messages, and smartphone apps have been effective in the treatment of obesity [16-19] and can address many of the limitations associated with in-person treatment [20]. By maintaining regular interaction with health care providers and directly receiving educational content, support, and motivation in a widely accessible, convenient, and affordable format, patient engagement and adherence are improved [17,21]. Although the evidence for clinical effectiveness continues to grow, the literature lacks sufficient data on health care cost savings from mHealth programs [22].

Noom (Noom Inc) is an mHealth program that delivers a comprehensive lifestyle intervention through educational articles, coaching, support groups, diet and exercise tracking, and techniques based on the principles of cognitive behavioral therapy. Noom has 2 health programs: Noom Weight for weight management and Noom Mood for stress management. Previous work has shown that 56% of Noom Weight starters achieve weight loss of ≥5% of initial body weight after 6 months [23], a threshold shown to produce clinically meaningful improvements in health by improving lipid profiles and reducing the risks of developing diabetes and hypertension [8,24]. A retrospective analysis of >11,000 users who opened the program at least once after week 8 showed that the majority achieved ≥5% weight loss at 32 weeks (79%) and 52 weeks (82%), with the proportion of users losing ≥10% body weight increasing from 30% to 40% over the same period [25]. The degree of weight loss achieved has also been demonstrated to be strongly associated with user engagement levels [16,25-29]. Although the clinical benefit of Noom Weight has been established, its economic impact, such as that on health care resource utilization (HRU) and associated costs, has not been thoroughly evaluated or reported in the literature to date.

### Objectives

We conducted a retrospective study using real-world data from the Noom Weight user database, electronic health records (EHRs), and a commercial insurance claims database to assess the impact of Noom Weight on HRU and health care costs for Noom Weight users in the United States. Using propensity score analyses, HRU and costs among Noom Weight users with overweight and obesity were compared with those of demographically similar non–Noom Weight users at 12 and 24 months of follow-up. We hypothesized that Noom Weight users would demonstrate lower HRU and health care costs than individuals who did not use Noom Weight. A secondary aim was to explore the potential correlation of observed impacts on HRU and costs with changes in obesity-related clinical outcomes.

## Methods

### Study Design

This retrospective longitudinal cohort study used a data set based on new Noom Weight registrants between July 31, 2018, and July 31, 2020, including self-reported demographic data recorded at the time of registration (eg, age, sex, height, and weight) and activity data (eg, body weight measurements, food intake, and physical activity) recorded longitudinally thereafter. This data set was linked with a cohort of patients across Eversana EHR and open insurance claims data, which included anonymized patient identifiers, vital signs, and health care provider visits with associated diagnoses and procedures for American patients, including those with commercial insurance, Medicare, and Medicaid coverage. Eversana’s EHR data set is an aggregation and standardization of EHR data into a common data model for >120 million American patients. The data are derived from >2000 outpatient or ambulatory health centers, >500 hospitals, >30 health systems (including academic medical centers), and >50 unique electronic medical record platform providers across all 50 states in the United States. All database records are statistically deidentified and certified to be fully compliant with the patient confidentiality requirements set forth in the Health Insurance Portability and Accountability Act of 1996. A Health Insurance Portability and Accountability Act–compliant health data privacy service (Datavant) was used to create anonymized encrypted codes, which allowed for data sets to be linked together without the use or exchange of identifiable information [30]. All linked data were deidentified, and an expert determination was completed before data were received for analysis. No identifiable Noom Weight user information was used or exchanged in this study. HRU, health care costs, and obesity-related clinical outcomes were compared between Noom Weight users and demographically similar non–Noom Weight users with overweight or obesity.

### Ethics Approval

Noom Weight user data were collected from the Noom Weight database with prior approval from the Advarra Institutional Review Board (Pro00017565).

### Cohorts

#### Noom Weight Users

Noom Weight users were required to have an initial treatment window of continuous Noom Weight use lasting at least 3 months. Continuous use was defined as opening the Noom Weight program at least once per week on average. Each user’s unique index date was defined as the first day of Noom Weight use in the treatment window. If users recorded multiple eligible 3-month treatment windows, the earliest eligible window was used. Users were required to be US residents aged between 18 and 80 years and have a baseline BMI of ≥25 kg/m^2^. A minimum of 12 months of medical records before and after the index date, as well as a minimum documented insurance claims activity of at least 1 claim in the 12-month pre–index date period and at least 1 claim in the 12-month post–index date period, were required. In addition, users included in the 24-month post–index date analysis were required to have a second claim in the 12- to 24-month window.

Users were excluded if they had a history of medical conditions that would significantly affect body weight or the ability to fully engage in a comprehensive lifestyle intervention during the study period, including AIDS, cancer (all types), end-stage organ failure, hemiplegia, paraplegia, uncontrolled HIV infection, pregnancy, or wasting syndrome. Patients were also excluded if they had surgeries or acute-onset conditions affecting body weight, including bariatric surgery and cerebrovascular disease, at any time before the study up until before the end of the initial 3-month treatment window. Comorbidities were identified in the EHR using International Classification of Diseases, Ninth Revision, Clinical Modification and International Classification of Diseases, Tenth Revision, Clinical Modification codes.

#### Non–Noom Weight Users

A control cohort of non–Noom Weight users otherwise meeting the aforementioned inclusion and exclusion criteria defined for Noom Weight users were also identified using EHR and insurance claims data. The index date for non–Noom Weight users was defined as the date of the first qualifying BMI (≥25 kg/m^2^) entry recorded in the EHR between July 31, 2018, and July 31, 2020.

### Inverse Probability of Treatment Weighting Analysis

#### Baseline Covariates

Baseline covariates, including BMI, sex, age, and US census region were derived from Noom data for Noom Weight users and from EHR data for non–Noom Weight users. The type of insurance coverage was derived from insurance claims data for both cohorts. Covariates were balanced between the cohorts using inverse probability of treatment weighting (IPTW) before analyses.

#### HRU Determination

HRU was determined from all submitted insurance claims for any service. Claims were categorized based on the recorded place of service and type of claim, including inpatient visits, length of inpatient stay (in days), outpatient visits (including the number of clinic, office, and outpatient hospital visits), telehealth visits, other or unknown visits, surgeries, total prescriptions, and obesity-specific prescriptions. Unique visits were counted as single events regardless of the extent of services rendered during the visit, and total prescriptions included the total count of all prescribed medications. For each service type, the number of uses per patient, as well as the number of uses per patient among only those patients with ≥1 use, were determined at 12 and 24 months after the index date.

#### Health Care Costs

Health care costs were determined based on remitted insurance claims and included all unique entries with valid Current Procedural Terminology codes, Healthcare Common Procedure Coding System codes, or the National Drug Code. In cases where remitted amounts were not available, costs were imputed using the median remitted amount for similarly coded claims, aggregated on the claimant’s insurance type, age group, sex, and state of residence. Prescription costs included only paid claims; submitted claims that were not reimbursed were excluded. Obesity-specific prescription costs included all medications approved for short-term or chronic weight management or those commonly prescribed off-label. Costs per patient were calculated at 12 and 24 months for each service type among all patients, as well as among only those patients with ≥1 use of each service type. All costs were reported in US dollars and adjusted for inflation to 2021 US dollars using the medical consumer price index inflation factors from the Federal Reserve Economic Data repository [31].

### Statistical Analysis

Propensity score matching was conducted with IPTW to balance the Noom Weight and non–Noom Weight cohorts with respect to age, sex, geographic region, insurance plan, and BMI. Stabilized weights for reweighting were generated with the average treatment effect as the estimand. Summary statistics were expressed as mean and SD for continuous variables and frequency and percentage for categorical variables. Standardized mean differences (SMDs) were used to confirm covariate balance, with absolute SMDs <0.10 indicating potential balance. Mean differences (MDs) between the cohorts at 12 and 24 months were reported for HRU and costs. Generalized linear models were used to report incidence rate ratios (IRRs) for each HRU service (using a Poisson distribution with a log link) and cost ratios (CRs) for the overall costs (using a gamma distribution with a log link). All analyses were conducted using R statistical software (version 3.6.1; R Foundation for Statistical Computing).

### Subgroup Analysis

Subgroup analyses were conducted by stratifying cohorts according to the diagnosis of type 2 diabetes (T2D; yes vs no), the diagnosis of hypertension (yes vs no), index date BMI (≥35 kg/m^2^ vs <35 kg/m^2^), Noom Weight use duration (≥6 mo vs <6 mo), and Noom Weight engagement level (high vs low). Engagement was classified as *high* if the Noom Weight program was opened ≥6 days per week on average and classified as *low* if opened <6 days per week during the initial 3-month treatment period.

## Results

### Patient Demographics

A total of 114,691 Noom Weight users were represented in all 3 linked data sources, of whom 78,375 (68.34%) had valid index dates. After exclusions for comorbidities and inclusion criteria for index date BMI, index date age, Noom Weight use, and insurance claims activity were applied, of the 78,375 Noom Weight users, 43,047 (54.92%) were included for the 12-month analyses and 14,141 (18.04%) for the 24-month analyses. A total of 107,519 non–Noom Weight users were identified in both EHR and insurance claims data, of whom 95,005 (88.36%) had valid index dates. All inclusion and exclusion criteria were met by non–Noom Weight users for the 12-month (14,587/95,005, 15.35%) and 24-month (6487/95,005, 6.83%) analyses.

The baseline demographics of the study population are shown in Table 1 before and after IPTW. Before IPTW, the unweighted mean ages at baseline were 51.6 (SD 12.0) years for Noom Weight users and 52.7 (SD 14.3) years for non–Noom Weight users (SMD −0.077), and 82.75% (35,622/43,047) of the Noom Weight users and 54.67% (7975/14,587) of the non–Noom Weight users were female (SMD −0.635). After IPTW (ie, the sample analyzed for the study), the mean ages were equivalent between the cohorts (Noom Weight users: 51.9, SD 12.1 years; non–Noom Weight users: 51.9, SD 13.8 years; SMD 0.001), and the proportions of female users were identical at 75.6% (proportion after weighting) for both Noom Weight users and non–Noom Weight users (SMD 0.000). All other covariates were also well balanced after IPTW, with the proportion of balanced covariates (absolute SMDs <0.10) increasing from 23% to 100%. Relevant comorbid conditions before weighting are presented in Table 1.

**Table 1:** Baseline demographics before and after inverse probability of treatment weighting.

		Before weighting				After weighting		
		Noom Weight users (n=43,047)	Non–Noom Weight users (n=14,587)	SMD^a^	Noom Weight users^b^ (n=40,334^c^)		Non–Noom Weight users^b^ (n=10,549^c^)	SMD
Age (years), mean (SD)		51.6 (12.0)	52.7 (14.3)	−0.077	51.9 (12.1)		51.9 (13.8)	0.001
**Sex, n (%)**								
	Female	35,622 (82.8)	7975 (54.7)	N/A^d^	N/A (75.6)		N/A (75.6)	N/A
	Male	7425 (17.2)	6612 (45.3)	−0.635	N/A (24.4)		N/A (24.4)	0.000
**Region, n (%)**								
	South	17,902 (41.6)	6860 (47)	−0.128	N/A (43)		N/A (43.2)	−0.002
	North Central	10,371 (24.1)	3801 (26.1)	−0.070	N/A (24.6)		N/A (24.8)	−0.001
	West	8738 (20.3)	2905 (19.9)	−0.013	N/A (20.1)		N/A (19.9)	0.003
	Northeast	6033 (14)	1020 (7)	0.213	N/A (12.2)		N/A (12.2)	0.001
	Unknown	3 (0)	1 (0)	0.160	N/A (0)		N/A (0)	0.000
**Insurance type, n (%)**								
	Commercial	32,726 (76)	8736 (59.9)	0.103	N/A (71.9)		N/A (71.9)	0.000
	Medicare	8267 (19.2)	4150 (28.4)	−0.218	N/A (21.6)		N/A (21.6)	−0.001
	Medicaid	1798 (4.2)	1620 (11.1)	−0.260	N/A (6)		N/A (5.9)	0.000
	Others	256 (0.6)	81 (0.6)	0.125	N/A (0.6)		N/A (0.5)	0.001
BMI (kg/m^2^), mean (SD)		33.1 (6.2)	32.5 (6.5)	0.100	33 (6.1)		33.1 (7)	−0.024

^a^SMD: standardized mean difference.

^b^Except for BMI and age, only percentages are reported for categorical variables after weighting to show the balance in distributions across the 2 cohorts.

^c^Effective sample sizes after weighting.

^d^N/A: not applicable.

### HRU Assessment

Noom Weight users had statistically significantly lower HRU than non–Noom Weight users in the majority of places of service in both the 12-month (Table 2) and 24-month (Table 3) follow-up periods. Most notably, at 12 months after the index date, the average number of outpatient visits per person was 3.83 (SD 6.76) among Noom Weight users compared with 4.61 (SD 7.26) among non–Noom Weight users (MD −0.78, 95% CI −0.93 to −0.62; IRR 0.83, 95% CI 0.80-0.86; *P*<.001) and at 24 months after the index date was 8.16 (SD 11.77) visits among Noom Weight users compared with 8.74 (SD 12.04) visits among non–Noom Weight users (MD −0.58, 95% CI −1.00 to −0.17; IRR 0.93, 95% CI 0.89-0.98; *P*<.001). Fewer inpatient visits were recorded for Noom Weight users at 12 months (MD −0.03, 95% CI −0.04 to −0.03; IRR 0.53, 95% CI 0.47-0.60; *P*<.001) and 24 months (MD −0.04, 95% CI −0.06 to −0.02; IRR 0.68, 95% CI 0.58-0.79; *P*<.001) after the index date, and fewer surgeries were recorded at 12 months (MD −0.01, 95% CI −0.00 to −0.01; IRR 0.44, 95% CI 0.34-0.56; *P*<001) and 24 months (MD −0.01, 95% CI −0.01 to 0.00; IRR 0.67, 95% CI 0.51-0.86; *P*=.004) after the index date. Noom Weight users also had fewer prescriptions than non–Noom Weight users at 12 months (MD −1.39, 95% CI −1.76 to −1.03; IRR 0.92, 95% CI 0.90-0.94; *P*<.001) and 24 months (MD −3.13, 95% CI −4.25 to −2.00; IRR 0.92, 95% CI 0.89-0.95; *P*<.001) after the index date. The number of obesity-specific prescriptions was slightly higher among Noom Weight users than among non–Noom Weight users at 12 months after the index date (MD 0.08, 95% CI 0.01-0.16; IRR 1.07, 95% CI 1.01-1.14; *P*=.03), as was the number of telehealth visits (MD 0.02, 95% CI 0.01-0.04; IRR 1.50, 95% CI 1.15-1.97; *P*=.003), although significant differences did not persist at 24 months after the index date (*P*=.53 and *P*=.51, respectively). Additional analyses limited to patients with at least 1 encounter of each service type showed lower outpatient service use at 12 months after the index date as well as fewer prescriptions at 12 and 24 months after the index date for Noom Weight users compared with non–Noom Weight users (Table S1 in Multimedia Appendix 1).

**Table 2:** Health care resource utilization rates by service type at 12 months after the index date.

Service type			Noom Weight users (n=40,334^a^), mean (SD)		Non–Noom Weight users (n=10,549^a^), mean (SD)		Comparison between cohorts						
							Mean difference (95% CI)		*P* value		Incidence rate ratio (95% CI)		*P* value
Inpatient visits			0.04 (0.27)		0.07 (0.40)		−0.03 (−0.04 to −0.03)		<.001		0.53 (0.47 to 0.60)		<.001
Inpatient days			0.08 (0.76)		0.15 (1.09)		−0.07 (−0.09 to −0.05)		<.001		0.52 (0.45 to 0.61)		<.001
Telehealth visits			0.07 (0.81)		0.05 (0.56)		0.02 (0.01 to 0.04)		<.001		1.50 (1.15 to 1.97)		.003
**Outpatient visits**													
	All	3.83 (6.76)		4.61 (7.26)		−0.78 (−0.93 to −0.62)		<.001		0.83 (0.80 to 0.86)		<.001	
	Clinic	0.17 (1.86)		0.18 (1.99)		−0.01 (−0.05 to 0.03)		.57		0.94 (0.77 to 1.15)		.57	
	Office	2.76 (5.55)		3.25 (5.75)		−0.49 (−0.61 to −0.37)		<.001		0.85 (0.82 to 0.88)		<.001	
	Hospital	0.90 (2.58)		1.17 (3.02)		−0.28 (−0.34 to −0.21)		<.001		0.77 (0.72 to 0.81)		<.001	
	Other visits^b^	0.86 (2.72)		0.95 (3.67)		−0.09 (−0.16 −0.01)		.02		0.91 (0.84 to 0.98)		.02	
	Surgeries	0.01 (0.09)		0.01 (0.14)		−0.01 (−0.01 to 0.00)		<.001		0.44 (0.34 to 0.56)		<.001	
**Prescriptions**													
	All	16.63 (15.73)		18.02 (17.23)		−1.39 (−1.76 to −1.03)		<.001		0.92 (0.90 to 0.94)		<.001	
	Obesity specific	1.21 (3.38)		1.12 (3.34)		0.08 (0.01 to 0.16)		.03		1.07 (1.01 to 1.14)		.03	

^a^Effective sample size.

^b^Includes visits of unlisted or unknown types.

**Table 3:** Health care resource utilization rates by service type at 24 months after the index date.

Service type		Noom Weight users (n=11,438^a^), mean (SD)	Non–Noom Weight users (n=4485^a^), mean (SD)	Comparison between cohorts			
				Mean difference (95% CI)	*P* value	Incidence rate ratio (95% CI)	*P* value
Inpatient visits		0.09 (0.51)	0.13 (0.55)	−0.04 (−0.06 to −0.02)	<.001	0.68 (0.58 to 0.79)	<.001
Inpatient days		0.20 (1.52)	0.29 (1.43)	−0.08 (−0.13 to −0.04)	<.001	0.71 (0.59 to 0.85)	<.001
Telehealth visits		0.14 (1.53)	0.13 (1.42)	0.02 (−0.04 to 0.07)	.51	1.15 (0.76 to 1.74)	.52
**Outpatient visits**							
	All	8.16 (11.77)	8.74 (12.04)	−0.58 (−1.00 to −0.17)	.006	0.93 (0.89 to 0.98)	.005
	Clinic	0.34 (2.62)	0.38 (2.58)	−0.03 (−0.12 to 0.06)	.48	0.92 (0.72 to 1.17)	.48
	Office	5.87 (9.57)	6.12 (9.61)	−0.25 (−0.59 to 0.09)	.14	0.96 (0.91 to 1.01)	.14
	Hospital	1.94 (4.71)	2.24 (4.69)	−0.30 (−0.46 to −0.14)	<.001	0.87 (0.80 to 0.94)	<.001
	Other visits^b^	1.80 (4.70)	1.71 (5.42)	0.09 (−0.08 to 0.27)	.31	1.05 (0.95 to 1.17)	.32
	Surgeries	0.02 (0.14)	0.02 (0.18)	−0.01 (−0.01 to 0.00)	.004	0.67 (0.51 to 0.86)	.002
**Prescriptions**							
	All	35.37 (30.47)	38.50 (32.62)	−3.13 (−4.25 to −2.00)	<.001	0.92 (0.89 to 0.95)	<.001
	Obesity specific	2.62 (6.50)	2.55 (6.59)	0.08 (−0.16 to 0.31)	.53	1.03 (0.94 to 1.13)	.53

^a^Effective sample size.

^b^Includes visits of unlisted or unknown types.

### HRU: Subgroup Analysis

When comparing Noom Weight users and non–Noom Weight users, subgroups without T2D or hypertension and with BMI <35 kg/m^2^ had lower use of more service types than subgroups with T2D or hypertension and with BMI ≥35 kg/m^2^, respectively (Tables S2-S4 in Multimedia Appendix 1); for example, although fewer outpatient visits were recorded among Noom Weight users than among non–Noom Weight users in both subgroups with and without T2D at 12 months after the index date, significant differences (reductions) were also observed for Noom Weight users compared with non–Noom Weight users in inpatient visits, inpatient days, surgeries, prescriptions, and obesity-specific prescriptions only in the subgroup without T2D (all *P*<.05; Table S2 in Multimedia Appendix 1). Similarly, relatively fewer significant differences between Noom Weight and non–Noom Weight users were observed for the subgroup with hypertension (all *P*<.05; Table S3 in Multimedia Appendix 1) and with BMI ≥35 kg/m^2^ (all *P*<.05; Table S4 in Multimedia Appendix 1) in the respective subgroup analyses. The differences between the subgroups were more pronounced at 24 months after the index date.

More than three-quarters (33,810/44,416, 76.12%) of the Noom Weight users were categorized as *high engaged*, with the remaining users (10,606/44,416, 23.88%) categorized as *low engaged*. High-engaged Noom Weight users had significantly fewer prescriptions (overall and obesity specific) than low-engaged users at 12 months (overall MD −0.95, 95% CI −1.40 to −0.50; obesity-specific MD −0.16, 95% CI −0.26 to −0.07) and at 24 months (overall MD −2.79, 95% CI −4.41 to −1.17; obesity-specific MD −0.52, 95% CI −0.86 to −0.18) after the index date, and both engagement levels had significantly fewer inpatient visits, inpatient days, outpatient visits, and prescriptions than non–Noom Weight users at 12 months after the index date (Table S5 in Multimedia Appendix 1). These differences remained significant at 24 months after the index date for high-engaged Noom Weight users; for low-engaged Noom Weight users, only the differences in inpatient visits and outpatient visits remained significant at 24 months after the index date, and increases in obesity-specific prescriptions among low-engaged Noom Weight users compared with non–Noom Weight users were also noted at 12 and 24 months after the index date.

The mean duration of Noom Weight use was 8.67 (SD 5.70) months among all Noom Weight users, with 46.22% (20,530/44,416) using Noom Weight for <6 months and 53.78% (23,888/44,416) using Noom Weight for ≥6 months. Noom Weight users with ≥6 months of use had fewer prescriptions than users with <6 months of use at 12 months after the index date (MD −0.64, 95% CI −1.04 to −0.24), but a significant difference did not persist at 24 months (Table S6 in Multimedia Appendix 1). The pattern of significant differences for Noom Weight users of both durations was similar to that for Noom Weight engagement level at 12 months after the index date, with fewer inpatient visits, inpatient days, outpatient visits, surgeries, and prescriptions than for non–Noom Weight users. This pattern of significant differences was unchanged at 24 months after the index date for Noom Weight users with ≥6 months of use; for Noom Weight users with <6 months of use, only inpatient visits, inpatient days, a subset of outpatient visits (outpatient hospital visits only), and prescriptions were significantly lower than those for non–Noom Weight users.

### Health Care Costs

Noom Weight users had lower overall health care costs at 12 months after the index date, with average expenditures of US $3433.89 (SD $10,397.96) per person compared with US $3884.28 (SD $13,661.66) per person for non–Noom Weight users (MD −450.39, 95% CI −706.28 to −194.50; CR 0.91, 95% CI 0.85-0.97; *P*<.001; Table 4). At 24 months, average overall costs for Noom Weight users were US $7367.97 (SD $19,748.80) per person compared with US $8587.03 (SD $29,190.01) per person for non–Noom Weight users (MD −1219.06, 95% CI −2061.56 to −376.55; CR 0.86, 95% CI 0.78-0.95; *P*=.005; Table 5). Expenditures for inpatient services, outpatient services, and overall prescriptions were lower for Noom Weight users than for non–Noom Weight users at 12 months, whereas telehealth expenditures were slightly higher. Of these, the reductions in outpatient expenditures, overall prescriptions, and overall costs remained statistically significant through 24 months. The additional analysis limited to patients with at least 1 encounter of each service type (Table S7 in Multimedia Appendix 1) showed significantly lower overall and obesity-specific prescription costs at both time points as well as significantly lower outpatient costs at 12 months for Noom Weight users compared with non–Noom Weight users (all *P*<.05).

**Table 4:** Health care costs by service type at 12 months after the index date.

Service type				Noom Weight users (US $; n=40,334^a^), mean (SD)		Non–Noom Weight users (US $; n=10,549^a^), mean (SD)		Mean difference (95% CI)		*P* value
Inpatient services				24.19 (368.01)		44.29 (497.70)		−20.10 (−30.08 to −10.12)		<.001
Telehealth services				6.08 (85.27)		3.52 (46.48)		2.56 (1.37 to 3.76)		<.001
**Outpatient services**										
		All	492.50 (1360.32)		616.83 (1779.75)		−124.33 (−159.76 to −88.89)		<.001	
		Clinic	16.03 (134.04)		16.25 (151.81)		−0.22 (−2.74 to 2.29)		.86	
		Office	268.33 (835.97)		348.77 (1167.06)		−80.43 (−103.24 to −57.63)		<.001	
		Hospital	208.14 (978.29)		251.81 (1193.71)		−43.67 (−68.23 to −19.11)		<.001	
		Other services^b^	47.61 (1117.32)		42.32 (243.35)		5.29 (−6.95 to 17.52)		.40	
**Prescriptions**										
	All		2863.51 (10,160.60)		3177.33 (13,482.12)		−313.82 (−565.42 to −62.21)		.02	
	Obesity-specific		430.81 (2997.12)		466.96 (3047.48)		−36.15 (−101.33 to 29.02)		.28	
Overall (all service types)^c^				3433.89 (10,397.96)		3884.28 (13,661.66)		−450.39 (−706.28 to −194.50)		<.001

^a^Effective sample size.

^b^Includes costs of unlisted or unknown types.

^c^The overall cost ratio was 0.91 (95% CI 0.85-0.97; *P*=.004), based on a gamma regression model and after cases with US $0 costs were removed.

**Table 5:** Health care costs by service type at 24 months after the index date.

Service type			Noom Weight users (US $; n=11,438^a^), mean (SD)		Non–Noom Weight users (US $; n=4485^a^), mean (SD)		Mean difference (95% CI)		*P* value
Inpatient services			54.65 (581.75)		62.62 (428.17)		−7.96 (−23.70 to 7.78)		.32
Telehealth services			14.04 (208.52)		9.06 (94.49)		4.97 (0.17 to 9.78)		.04
**Outpatient services**									
	All	999.78 (2148.88)		1080.37 (2061.25)		−80.60 (−151.10 to −10.09)		.03	
	Clinic	35.10 (239.53)		38.80 (307.65)		−3.70 (−11.97 to 4.57)		.38	
	Office	572.34 (1448.81)		598.52 (1399.89)		−26.17 (−71.92 to 19.57)		.26	
	Hospital	392.33 (1335.99)		443.05 (1250.56)		−50.72 (−95.66 to −5.79)		.03	
	Other services^b^	84.43 (477.39)		80.38 (515.89)		4.05 (−8.55 to 16.65)		.53	
**Prescriptions**									
	All	6215.07 (19,439.55)		7354.59 (28,966.20)		−1139.52 (−1972.21 to −306.83)		.007	
	Obesity specific	918.06 (5578.53)		1149.87 (6679.35)		−231.81 (−459.45 to −4.16)		.05	
Overall (all service types)^c^			7367.97 (19,748.80)		8587.03 (29,190.01)		−1219.06 (−2061.56 to −376.55)		.005

^a^Effective sample size.

^b^Includes costs of unlisted or unknown types.

^c^The overall cost ratio was 0.86 (95% CI 0.78-0.95; *P*=.004), based on a gamma regression model and after cases with US $0 costs were removed.

### Health Care Costs: Subgroup Analysis

The results of the subgroup analyses for costs showed patterns similar to those for HRU. Overall, significantly lower costs were seen for Noom Weight users compared with non–Noom Weight users in more service types among cases without T2D (vs cases with T2D), without hypertension (vs cases without hypertension), and with BMI <35 kg/m^2^ (vs cases with BMI ≥35 kg/m^2^; all *P*<.05; Tables S8-S10 in Multimedia Appendix 1). Despite lower HRU among high-engaged versus low-engaged Noom Weight users and Noom Weight users with longer versus shorter duration of use, no significant corresponding differences in costs were observed between these groups (Tables S11 and S12 in Multimedia Appendix 1). Compared with non–Noom Weight users, high-engaged Noom Weight users had significantly lower costs at 12 months after the index date for inpatient and outpatient visits as well as lower overall costs, and significantly lower prescription costs and overall costs at 24 months after the index date. Low-engaged Noom Weight users had fewer differences in costs compared with non–Noom Weight users, with significantly lower costs for inpatient and outpatient visits as well as lower overall costs at 12 months after the index date, and no significant differences at 24 months after the index date. The pattern of significant cost differences for Noom Weight use ≥6 months and Noom Weight use <6 months compared with non–Noom Weight users was similar to that for high-engaged Noom Weight users and low-engaged Noom Weight users, respectively, at 12 and 24 months after the index date.

## Discussion

### Principal Findings

We showed that HRU is lower for Noom Weight users than for non–Noom Weight users at 12 and 24 months after the index date. Per user, 0.03 fewer inpatient visits, 0.83 fewer outpatient visits, 0.01 fewer surgeries, and 1.39 fewer prescriptions were recorded among Noom Weight users compared with non–Noom Weight users at 12 months after the index date. At 24 months after the index date, 0.04 fewer inpatient visits, 0.58 fewer outpatient visits, 0.01 fewer surgeries, and 3.13 fewer prescriptions were recorded among Noom Weight users compared with non–Noom Weight users. Noom Weight users had higher use of telehealth services at 12 months after the index date (MD 0.02/user), perhaps because of increased connectivity to digital health services owing to their use of Noom Weight or because of increased health responsibility as a result of the program [32]. There were also a greater number of obesity-specific prescriptions for Noom Weight users compared with non–Noom Weight users at 12 months (MD 0.08/user), which may be related to more health-conscious behavior [32] among newly registered Noom Weight users, potentially leading to higher rates of prescriptions. A statistically significant difference did not persist at 24 months.

The results also showed significantly lower health care costs for Noom Weight users compared with non–Noom Weight users at both 12 months and 24 months after the index date. Overall costs for Noom Weight users were US $450 lower per person at 12 months and US $1219 lower per person at 24 months compared with overall costs for individuals who did not use Noom Weight. Furthermore, extending similar findings at 12 months, outpatient services costs (MD US $80/person) and prescription costs (MD US $1139/person) were lower for Noom Weight users than for non–Noom Weight users at 24 months after the index date.

Overall, our findings demonstrate significantly lower HRU and costs at 12 and 24 months for Noom Weight users compared with demographically similar non–Noom Weight users, with greater impact on HRU and costs observed for Noom Weight users without T2D, without hypertension, with BMI <35 kg/m^2^, with higher Noom Weight engagement, and with longer duration of Noom Weight use.

### Limitations

This was an observational study, which therefore does not permit causal associations to be drawn between Noom Weight use and HRU and cost outcomes. Another important limitation was the restricted sample size owing to the linking of 3 separate databases. Users were required to be present in all 3 data sources for inclusion, which sharply reduced the size of the available population. This also adds a risk of bias because the underlying systematic exclusions owing to missing data that may have affected patients in any 1 database would have been projected across all 3 databases, including those not previously affected by them. The requirement for Noom Weight users to use the program for 3 months may have biased this cohort toward including more health-conscious and motivated users, although it should be noted that this engagement criterion is similar to that used for previously studied Noom Weight populations and that this study’s inclusion requirement to use the program at least 10 times in total during this time period is relatively low [25-27,29,33,34]. In addition, this study included only US residents, which may limit its generalizability. However, previous work has shown the comparable effectiveness of Noom Weight use for weight loss across different regions and income levels [16,28]. This may suggest similar cross-national effects on HRU and costs, which would be more affected by access to health care and existing HRU patterns in each country than by the differential impact of Noom Weight use. Furthermore, the study cohorts described here included mostly women and comprised individuals aged <80 years, further limiting the generalizability of the results.

Some potential imbalances between the cohorts may not have been accounted for in our IPTW analyses. Potential racial imbalances could not be accounted for because the Noom Weight user and non–Noom Weight user cohorts had either nonspecific or missing information for race, which prevented reweighting on this variable. Preexisting comorbidities were also not included in reweighting to permit subgroup analyses based on comorbid conditions. However, these were nevertheless reasonably well balanced in the reweighted cohorts. There may also be other confounding variables affecting HRU and costs that were not identified or accounted for in our analyses (eg, education level and income bracket). The potential impact of other common weight loss interventions used concurrently, such as weight loss programs and antiobesity medications, on the study results also requires further investigation.

In particular, an important limitation is some concurrent use of antiobesity medications, which raises the question of whether the effects were driven by the use of medications or the use of Noom Weight; this question cannot be definitively answered by this study. The data suggest that it is unlikely that this was a confound that primarily drove the results observed because there was no significant difference in antiobesity medication use between the Noom Weight user and non–Noom Weight user groups at 24 months after the index date despite significant differences in other types of HRU and costs, and a subgroup analysis of individuals with at least 1 prescription or health care visit showed no difference in obesity-specific prescriptions between the groups at all time points. However, any impact of antiobesity medications is not measured or ruled out by this study. Future research, especially with causal designs, should elucidate when and to what magnitude the concurrent use of antiobesity medications influences other types of HRU and costs. Furthermore, the study excluded individuals who had bariatric or cerebrovascular disease at any time before the study up until before the end of the initial 3-month treatment window. Future studies should examine how receiving bariatric surgery concurrently with Noom Weight use affects HRU and costs, especially on longer time scales, and whether the use of Noom Weight could be associated with motivation to undergo bariatric surgery.

Open insurance claims data were used, which allowed the assessment of direct medical costs in all care settings (eg, inpatient and outpatient) and provided large sample sizes covering patients with diverse backgrounds and medical needs. However, there are limitations associated with the use of open claims data. Open claims databases effectively capture patient activity longitudinally, but they do not necessarily capture all patient claims activity within a given time period. HRU involving service providers not included in the database will not be captured, giving a potentially incomplete picture of HRU and costs, biasing the results if certain types of HRU are less well represented and potentially excluding otherwise eligible patients for claims inactivity if they have unobserved claims. We applied a minimum claim activity criterion of 1 claim per 12-month period during the study period to mimic a continuous enrollment criterion that would be applied to a closed claims data set. Although this was a low threshold that preserved sample size, it may have introduced some bias toward patients more likely to file claims and therefore patients who were potentially sicker. As not all submitted claims in open claims databases are remitted, missing values were imputed to estimate costs. Imputation may potentially over- or underestimate true costs and systematically bias any subcategory of HRU that is particularly affected by missing data. Finally, because open claims databases are based on a large convenience sample that is not random, there may be potential biases or issues with generalizability.

Discrete surgical visits could be readily determined from insurance claims data for HRU analyses. However, individual Current Procedural Terminology codes for activities within each surgery were frequently not available and were aggregated under a master code for the entire procedure. This prevented meaningful cost assessments for surgeries, which would require the enumeration of the specific line items, and therefore surgical costs were only captured as a subset of overall costs.

### Comparison With Prior Work

Noom Weight has previously been shown to be an effective treatment for obesity, frequently producing weight loss exceeding 5% of initial body weight [23,27,34,35] in as little as 8 weeks [36] and persisting for up to 52 weeks [25]. However, Noom Weight’s impact and the impact of mHealth technologies generally on HRU and costs among users with overweight or obesity compared with a demographically similar control group have not been previously reported. Therefore, this study contributes to filling a substantial gap in the literature regarding the limited data on health care costs and HRU associated with digital programs. In the following paragraphs, we compare these findings to those of the few publications reporting on the impact of nonsurgical (ie, behavioral, not including bariatric surgery) weight loss on HRU and costs.

One study compared health care costs over 3 years for 4790 users of an employer-sponsored digital weight loss program with those for a propensity-matched control group (n=4790) who did not use the program [37]. Overall costs for those who used the program were US $771 lower per person over 3 years compared with those for nonusers. Specifically, the program was associated with lower outpatient (US $609/person) and inpatient (US $162/person) costs over 3 years. Our results compare favorably, showing even greater cost savings because, compared with non–Noom Weight users, Noom Weight users had lower overall costs of US $1219 per person over 2 years. In the study by Horstman et al [37], cost savings were mostly concentrated in outpatient costs, whereas we found comparatively lower outpatient cost savings over 2 years (US $78/person). This could be because of the limited availability of EHR data in our study in contrast to the health plan data used in the study by Horstman et al [37]. Future studies should collect full-scale health and insurance plan data in addition to the open insurance claims data used in this study.

An investigation by Ding et al [38] on the impact of nonsurgical weight loss on health care costs used insurance claims and EHR data for 20,488 adults with obesity in the IBM MarketScan Explorys Claims-Electronic Medical Record Data Set and found statistically significantly reduced costs for patients with >5% weight loss compared with those maintaining steady weight [38]. This aligns with our finding that Noom Weight users who were in this dedicated weight management program exhibited lower costs than the non–Noom Weight group. Furthermore, Ding et al [38] reported smaller absolute cost reductions after 2 years than in the first year alone, although the study did not directly compare the magnitude and significance of costs from 1 year to 2 years. In this study, cost reductions increased from year 1 through year 2 in absolute value. Although the 2 studies are not directly comparable, this raises the possibility that the cost impact of Noom Weight use may be longer lasting than that observed with nonsurgical weight loss interventions in general. This is consistent with the typical trend of long-term weight regain (potentially correlating with increased costs) among those with nonsurgical weight loss in the absence of intensive lifestyle interventions such as Noom Weight [8]. Future research should test this explanation.

The degree of weight loss among Noom Weight users is closely tied to the level of user engagement, with greater weight loss among patients who more frequently read articles, log data, and interact with coaches [25,27]. Similar results have also been reported with other weight loss programs [39]. In our study, higher Noom Weight engagement was also associated with lower HRU in terms of the number of prescriptions claimed (Table 3). High-engaged Noom Weight users claimed 0.95 (almost 1 unit) fewer prescriptions than low-engaged Noom Weight users through 12 months, increasing to 2.79 fewer prescriptions through 24 months. This was also true for obesity-specific prescriptions, which were fewer for high-engaged Noom Weight users than for low-engaged Noom Weight users at 12 months (MD −0.16) and 24 months (MD −0.52). Although this did not translate into statistically significantly lower costs for high-engaged Noom Weight users compared with low-engaged Noom Weight users, high Noom Weight engagement was associated with statistically significantly lower overall costs of −US $462 per user (95% CI −US $775.62 to −US $148.39) compared with non–Noom Weight use at 12 months, as well as lower overall costs of −US $1446 (95% CI −US $2469 to −US $422) and lower prescription costs of −US $1366 (95% CI −US $2377 to −US $355) compared with non–Noom Weight use at 24 months (Table S4 in Multimedia Appendix 1). In comparison, costs for these service types were not statistically significantly different for low-engaged Noom Weight users compared with non–Noom Weight users at 12 or 24 months.

In addition to subgroup analyses based on Noom Weight engagement, we also performed subgroup analyses according to BMI (<35 kg/m^2^ vs ≥35 kg/m^2^), T2D diagnosis, and hypertension diagnosis. Costs were significantly lower for the Noom Weight group versus the non–Noom Weight group for more HRU types in samples without T2D (vs samples with T2D), without hypertension (vs samples with hypertension), and with BMI <35 kg/m^2^ (vs samples with BMI ≥35 kg/m^2^). This could be because these conditions incur substantial health care costs; for example, poor glycemic control, as seen in T2D, is related to higher total health care, hospitalization, and medication costs [40]; in another study, the presence of hypertension substantially increased health care costs [41]. Therefore, cost differences between the Noom Weight user and non–Noom Weight user groups are likely starker without these conditions than with these conditions.

Previous work suggests some potential mechanisms for the results found in this study. We speculate that Noom Weight users may have shown significant cost savings compared with non–Noom Weight users because weight management efforts reduced the incidence of chronic conditions and their associated medical costs [42-44]. We also speculate that Noom Weight’s educational content on healthy behaviors could result in improved medication adherence, especially because a previous study found that Noom Weight users’ health responsibility (eg, taking interest in, and responsibility for, their overall physical health) improved over the course of the program [32,45]. However, because this study did not test causal pathways, future research should test and identify potential mechanisms.

### Conclusions

To our knowledge, this is the first study using real-world data to show the economic impact of the use of an mHealth intervention by a cohort of users with overweight and obesity compared with a control cohort not using the mHealth intervention. We show lower HRU and costs for users of the Noom Weight mHealth program compared with non–Noom Weight users over a 2-year follow-up period. Comprehensively examining all service types, we found that inpatient visits, outpatient visits, surgical visits, and prescriptions were lower for Noom Weight users than for non–Noom Weight users for up to 24 months after initiating Noom Weight. Costs per Noom Weight user were statistically significantly lower by US $80 for outpatient services, US $1139 for prescriptions, and US $1219 overall at 24 months, which could correspond to savings of approximately US $609 per person per year during this period. These cost estimates compare favorably with those of previously studied programs. By linking Noom Weight data, EHR data, and insurance claims data, we were able to conduct several subgroup analyses for HRU and costs, including analyses based on T2D diagnosis or hypertension diagnosis, duration of Noom Weight use, user engagement level, and index date BMI. Further research is required to establish the relationship between changes in weight and BMI, as well as in comorbidities, with changes in HRU and costs, including the impact of the differential levels of weight loss. In addition, because this study focused on direct health care costs only, future research should investigate the impact of mHealth interventions on indirect costs (eg, productivity costs) as well.

## Appendix

Multimedia Appendix 1 Tables displaying results from subgroup analyses.

## Abbreviations

CR: cost ratio
EHR: electronic health record
HRU: health care resource utilization
IPTW: inverse probability of treatment weighting
IRR: incidence rate ratio
MD: mean difference
mHealth: mobile health
SMD: standardized mean difference
T2D: type 2 diabetes
