# Supplementary material for: The Impact of a Digital Weight Loss Intervention on Health Care Resource Utilization and Costs Compared Between Users and Nonusers With Overweight and Obesity: Retrospective Analysis Study
Source: JMIR Mhealth Uhealth. 2023 Aug 24;11:e47473. doi: 10.2196/47473 (PMC10485704; doi:10.2196/47473)
Supplement: Multimedia Appendix 1 [file mhealth_v11i1e47473_app1.docx]

**Table S1**. Healthcare resource utilization rates by service type among patients with at least 1 encounter

| Service type | | Noom users | Non-Noom users | Comparison between cohorts | | | |
| --- | --- | --- | --- | --- | --- | --- | --- |
|  |  | Mean (SD) | Mean (SD) | Mean difference (95% CI) | P value | Incidence rate ratio (95% CI) | P value |
| **12 months post-index^a^** | | | | | | | |
| Inpatient visits | | 1.27 (0.93) | 1.31 (1.14) | -0.04 (-0.14, 0.06) | 0.417 | 0.97 (0.90, 1.05) | 0.415 |
| Inpatient days | | 2.74 (3.51) | 2.86 (3.79) | -0.13 (-0.47, 0.22) | 0.475 | 0.96 (0.85, 1.08) | 0.473 |
| Telehealth visits | | 2.66 (4.31) | 2.27 (3.21) | 0.39 (-0.13, 0.91) | 0.142 | 1.17 (0.94, 1.46) | 0.162 |
| **Outpatient visits** | |  |  |  |  |  |  |
|  | All | 5.49 (7.51) | 5.98 (7.76) | -0.49 (-0.68, -0.30) | <0.001^b^ | 0.92 (0.89, 0.95) | <0.001^b^ |
|  | Clinic | 1.93 (5.93) | 2.36 (6.79) | -0.43 (-0.85, -0.01) | 0.043^b^ | 0.82 (0.68, 0.99) | 0.035^b^ |
|  | Office | 4.85 (6.63) | 5.13 (6.52) | -0.28 (-0.46, -0.10) | 0.002^b^ | 0.95 (0.91, 0.98) | 0.002^b^ |
|  | Hospital | 2.69 (3.89) | 2.89 (4.18) | -0.20 (-0.34, -0.06) | 0.005^b^ | 0.93 (0.89, 0.98) | 0.005^b^ |
| Other/unknown visits | | 3.05 (4.42) | 3.21 (6.19) | -0.16 (-0.39, 0.06) | 0.156 | 0.95 (0.88, 1.02) | 0.148 |
| Surgeries | | 1.10 (0.45) | 1.16 (0.59) | -0.06 (-0.17, 0.05) | 0.287 | 0.95 (0.86, 1.04) | 0.283 |
| **Prescriptions** | |  |  |  |  |  |  |
|  | All | 17.66 (15.64) | 19.17 (17.14) | -1.51 (-1.88, -1.13) | <0.001^b^ | 0.92 (0.90, 0.94) | <0.001^b^ |
|  | Obesity-specific | 6.43 (5.22) | 6.42 (5.46) | 0.01 (-0.28, 0.29) | 0.966 | 1.00 (0.96, 1.05) | 0.966 |
|  |  |  |  |  |  |  |  |
| **24 months post-index^a^** | | | | | | | |
| Inpatient visits | | 1.38 (1.53) | 1.43 (1.22) | -0.05 (-0.19, 0.10) | 0.503 | 0.97 (0.87, 1.07) | 0.503 |
| Inpatient days | | 3.23 (5.18) | 3.20 (3.65) | 0.03 (-0.44, 0.50) | 0.887 | 1.01 (0.87, 1.17) | 0.887 |
| Telehealth visits | | 3.45 (6.67) | 3.09 (6.38) | 0.36 (-0.84, 1.57) | 0.554 | 1.12 (0.77, 1.63) | 0.565 |
| **Outpatient visits** | |  |  |  |  |  |  |
|  | All | 9.49 (12.19) | 9.85 (12.35) | -0.36 (-0.81, 0.10) | 0.122 | 0.96 (0.92, 1.01) | 0.119 |
|  | Clinic | 2.37 (6.52) | 2.71 (6.45) | -0.35 (-0.92, 0.23) | 0.237 | 0.87 (0.70, 1.09) | 0.237 |
|  | Office | 8.04 (10.39) | 8.19 (10.32) | -0.15 (-0.57, 0.27) | 0.484 | 0.98 (0.93, 1.03) | 0.482 |
|  | Hospital | 3.87 (6.06) | 4.00 (5.68) | -0.13 (-0.40, 0.15) | 0.366 | 0.97 (0.90, 1.04) | 0.363 |
| Other/unknown visits | | 4.31 (6.49) | 4.17 (7.84) | 0.14 (-0.25, 0.53) | 0.486 | 1.03 (0.94, 1.13) | 0.490 |
| Surgeries | | 1.07 (0.30) | 1.15 (0.41) | -0.08 (-0.17, 0.01) | 0.067 | 0.93 (0.86, 1.00) | 0.064 |
| **Prescriptions** | |  |  |  |  |  |  |
|  | All | 36.15 (30.34) | 39.35 (32.47) | -3.19 (-4.32, -2.06) | <0.001^b^ | 0.92 (0.89, 0.95) | <0.001^b^ |
|  | Obesity-specific | 10.32 (9.33) | 10.47 (9.79) | -0.15 (-0.84, 0.55) | 0.678 | 0.99 (0.92, 1.05) | 0.677 |

^a^Sample sizes differ by service type.

^b^Significant at a .05 level

**Table S2**. Healthcare resource utilization rates by service type according to type 2 diabetes status

| Service type | | Diabetic | | | Non-diabetic | | |
| --- | --- | --- | --- | --- | --- | --- | --- |
|  |  | Noom users, mean (SD) | Non-Noom users, mean (SD) | Mean difference (95% CI) | Noom users, mean (SD) | Non-Noom users, mean (SD) | Mean difference, (95% CI) |
| 12 months post-index | | ESS = 2,200 | ESS = 992 |  | ESS = 38,154 | ESS = 9,570 |  |
| Inpatient visits | | 0.08 (0.47) | 0.11 (0.45) | -0.03 (-0.07, 0.00) | 0.03 (0.25) | 0.07 (0.39) | -0.03 (-0.04, -0.02)^b^ |
| Inpatient days | | 0.24 (1.69) | 0.29 (1.42) | -0.05 (-0.17, 0.06) | 0.07 (0.65) | 0.14 (1.06) | -0.07 (-0.09, -0.05)^b^ |
| Telehealth visits | | 0.08 (0.85) | 0.05 (0.59) | 0.03 (-0.02, 0.08) | 0.07 (0.81) | 0.04 (0.55) | 0.02 (0.01, 0.04)^b^ |
| **Outpatient visits** | |  |  |  |  |  |  |
|  | All | 5.77 (8.69) | 6.50 (9.60) | -0.73 (-1.40, -0.05)^b^ | 3.71 (6.60) | 4.43 (6.98) | -0.73 (-0.88, -0.57)^b^ |
|  | Clinic | 0.32 (3.62) | 0.42 (4.72) | -0.11 (-0.42, 0.20) | 0.16 (1.68) | 0.16 (1.51) | 0.00 (-0.03, 0.03) |
|  | Office | 3.95 (6.39) | 4.22 (6.32) | -0.26 (-0.72, 0.20) | 2.68 (5.48) | 3.16 (5.69) | -0.48 (-0.61, -0.35)^b^ |
|  | Hospital | 1.50 (3.47) | 1.85 (4.74) | -0.35 (-0.69, -0.02)^b^ | 0.86 (2.51) | 1.11 (2.80) | -0.25 (-0.31, -0.19)^b^ |
| Other/unknown visits | | 1.62 (4.03) | 1.83 (6.46) | -0.21 (-0.65, 0.23) | 0.81 (2.60) | 0.87 (3.28) | -0.05 (-0.12, 0.01) |
| Surgeries | | 0.02 (0.16) | 0.02 (0.23) | -0.01 (-0.02, 0.01) | 0.01 (0.08) | 0.01 (0.13) | -0.01 (-0.01, -0.00)^b^ |
| **Prescriptions** | |  |  |  |  |  |  |
|  | All | 27.06 (21.19) | 27.80 (22.13) | -0.74 (-2.40, 0.92) | 15.97 (15.08) | 17.12 (16.43) | -1.16 (-1.52, -0.79)^b^ |
|  | Obesity-specific | 4.02 (6.01) | 3.71 (5.94) | 0.31 (-0.14, 0.76) | 1.03 (3.06) | 0.89 (2.88) | 0.14 (0.07, 0.21)^b^ |
|  | |  |  |  |  |  |  |
| 24 months post-index | | ESS = 495 | ESS = 386 |  | ESS = 10,972 | ESS = 4,100 |  |
| Inpatient visits | | 0.15 (0.47) | 0.21 (0.84) | -0.06 (-0.13, 0.02) | 0.08 (0.51) | 0.12 (0.51) | -0.04 (-0.06, -0.02)^b^ |
| Inpatient days | | 0.37 (1.43) | 0.55 (2.09) | -0.18 (-0.40, 0.03) | 0.19 (1.52) | 0.26 (1.35) | -0.07 (-0.12, -0.02)^b^ |
| Telehealth visits | | 0.10 (0.61) | 0.10 (0.89) | 0.01 (-0.10, 0.12) | 0.15 (1.57) | 0.13 (1.46) | 0.02 (-0.04, 0.08) |
| **Outpatient visits** | |  |  |  |  |  |  |
|  | All | 12.04 (13.69) | 11.42 (13.85) | 0.63 (-1.22, 2.47) | 7.94 (11.62) | 8.50 (11.84) | -0.56 (-0.98, -0.13)^b^ |
|  | Clinic | 0.33 (1.53) | 0.64 (5.26) | -0.31 (-0.86, 0.24) | 0.34 (2.67) | 0.35 (2.17) | -0.01 (-0.09, 0.07) |
|  | Office | 8.66 (11.11) | 7.61 (9.81) | 1.04 (-0.31, 2.40) | 5.72 (9.45) | 5.99 (9.58) | -0.27 (-0.62, 0.07) |
|  | Hospital | 3.06 (5.94) | 3.16 (6.60) | -0.11 (-1.00, 0.78) | 1.88 (4.62) | 2.16 (4.47) | -0.28 (-0.44, -0.12)^b^ |
| Other/unknown visits | | 3.34 (6.26) | 2.51 (5.34) | 0.83 (0.15, 1.51)^b^ | 1.72 (4.59) | 1.64 (5.42) | 0.08 (-0.11, 0.26) |
| Surgeries | | 0.03 (0.21) | 0.03 (0.22) | -0.01 (-0.03, 0.02) | 0.02 (0.13) | 0.02 (0.17) | -0.01 (-0.01, -0.00)^b^ |
| **Prescriptions** | |  |  |  |  |  |  |
|  | All | 55.59 (37.47) | 54.49 (38.47) | 1.09 (-4.00, 6.19) | 34.23 (29.62) | 37.05 (31.65) | -2.82 (-3.95, -1.69)^b^ |
|  | Obesity-specific | 8.26 (11.13) | 7.16 (10.71) | 1.10 (-0.36, 2.57) | 2.30 (5.98) | 2.13 (5.91) | 0.17 (-0.05, 0.39) |

^a^ESS: Effective sample size

^b^Significant at a .05 level

**Table S3**. Healthcare resource utilization rates by service type according to hypertension status

| Service type | | Hypertensive | | | Non-hypetensive | | |
| --- | --- | --- | --- | --- | --- | --- | --- |
|  |  | Noom users, mean (SD) | Non-Noom users, mean (SD) | Mean difference (95% CI) | Noom users, mean (SD) | Non-Noom users, mean (SD) | Mean difference, (95% CI) |
| **12 months post-index** | | ESS = 7,256 | ESS = 2,594 |  | ESS = 33,119 | ESS = 7,981 |  |
| Inpatient visits | | 0.06 (0.37) | 0.10 (0.47) | -0.04 (-0.06, -0.02)^b^ | 0.03 (0.23) | 0.06 (0.37) | -0.03 (-0.04, -0.02)^b^ |
| Inpatient days | | 0.14 (1.14) | 0.21 (1.16) | -0.07 (-0.12, -0.02)^b^ | 0.07 (0.63) | 0.14 (1.07) | -0.07 (-0.09, -0.05)^b^ |
| Telehealth visits | | 0.07 (0.70) | 0.06 (0.63) | 0.01 (-0.02, 0.05) | 0.07 (0.83) | 0.04 (0.53) | 0.03 (0.01, 0.04)^b^ |
| **Outpatient visits** | |  |  |  |  |  |  |
|  | All | 5.30 (7.97) | 5.87 (8.13) | -0.57 (-0.92, -0.22)^b^ | 3.48 (6.39) | 4.24 (6.94) | -0.76 (-0.93, -0.59)^b^ |
|  | Clinic | 0.21 (2.39) | 0.21 (2.61) | 0.00 (-0.10, 0.10) | 0.16 (1.70) | 0.17 (1.78) | -0.01 (-0.05, 0.03) |
|  | Office | 3.71 (6.17) | 4.00 (6.05) | -0.28 (-0.55, -0.01)^b^ | 2.53 (5.37) | 3.03 (5.64) | -0.50 (-0.64, -0.36)^b^ |
|  | Hospital | 1.38 (3.36) | 1.66 (3.64) | -0.29 (-0.44, -0.13)^b^ | 0.78 (2.34) | 1.03 (2.80) | -0.25 (-0.31, -0.18)^b^ |
| Other/unknown visits | | 1.25 (3.39) | 1.38 (5.28) | -0.12 (-0.33, 0.08) | 0.77 (2.52) | 0.82 (3.04) | -0.06 (-0.13, 0.02) |
| Surgeries | | 0.01 (0.13) | 0.02 (0.16) | -0.01 (-0.02, 0.00) | 0.00 (0.07) | 0.01 (0.13) | -0.01 (-0.01, 0.00) |
| **Prescriptions** | |  |  |  |  |  |  |
|  | All | 21.55 (18.08) | 22.60 (18.82) | -1.05 (-1.88, -0.21)^b^ | 15.46 (14.88) | 16.70 (16.51) | -1.24 (-1.64, -0.84)^b^ |
|  | Obesity-specific | 1.52 (3.91) | 1.44 (3.90) | 0.08 (-0.10, 0.25) | 1.13 (3.24) | 1.03 (3.16) | 0.10 (0.02, 0.18)^b^ |
|  |  |  |  |  |  |  |  |
| **24 months post-index** | | ESS = 1,576 | ESS = 991 |  | ESS = 9,904 | ESS = 3,495 |  |
| Inpatient visits | | 0.14 (0.53) | 0.16 (0.58) | -0.02 (-0.06, 0.02) | 0.08 (0.51) | 0.12 (0.54) | -0.04 (-0.06, -0.02)^b^ |
| Inpatient days | | 0.36 (1.97) | 0.34 (1.31) | 0.02 (-0.10, 0.14) | 0.18 (1.42) | 0.27 (1.46) | -0.10 (-0.15, -0.05)^b^ |
| Telehealth visits | | 0.09 (0.91) | 0.12 (1.45) | -0.03 (-0.14, 0.07) | 0.15 (1.62) | 0.13 (1.42) | 0.03 (-0.03, 0.09) |
| **Outpatient visits** | |  |  |  |  |  |  |
|  | All | 11.24 (14.16) | 10.69 (13.33) | 0.55 (-0.47, 1.58) | 7.58 (11.17) | 8.20 (11.60) | -0.62 (-1.08, -0.17)^b^ |
|  | Clinic | 0.37 (2.59) | 0.34 (2.60) | 0.03 (-0.13, 0.19) | 0.34 (2.63) | 0.38 (2.57) | -0.05 (-0.15, 0.05) |
|  | Office | 8.09 (11.14) | 7.34 (10.33) | 0.75 (-0.07, 1.57) | 5.46 (9.19) | 5.79 (9.37) | -0.33 (-0.70, 0.04) |
|  | Hospital | 2.78 (5.91) | 3.00 (5.55) | -0.23 (-0.64, 0.18) | 1.79 (4.43) | 2.03 (4.41) | -0.24 (-0.42, -0.07)^b^ |
| Other/unknown visits | | 2.50 (5.52) | 2.26 (6.96) | 0.24 (-0.24, 0.71) | 1.67 (4.52) | 1.56 (4.90) | 0.11 (-0.07, 0.30) |
| Surgeries | | 0.02 (0.16) | 0.04 (0.23) | -0.02 (-0.03, 0.00) | 0.02 (0.13) | 0.02 (0.16) | -0.01 (-0.01, 0.00) |
| **Prescriptions** | |  |  |  |  |  |  |
|  | All | 45.45 (34.75) | 45.74 (33.19) | -0.29 (-2.95, 2.37) | 33.48 (29.21) | 36.49 (32.18) | -3.01 (-4.25, -1.78)^b^ |
|  | Obesity-specific | 3.36 (7.60) | 3.11 (7.25) | 0.25 (-0.34, 0.84) | 2.48 (6.27) | 2.39 (6.39) | 0.09 (-0.16, 0.35) |

^a^ESS: Effective sample size

^b^Significant at a .05 level

**Table S4**. Healthcare resource utilization rates by service type according to index BMI

| Service type | | Index BMI ≥ 35 | | | Index BMI < 35 | | |
| --- | --- | --- | --- | --- | --- | --- | --- |
|  |  | Noom users, Mean (SD) | Non-Noom users, Mean (SD) | Mean difference (95% CI) | Noom users, Mean (SD) | Non-Noom users, Mean (SD) | Mean difference, (95% CI) |
| **12 months post-index** | | ESS = 12,643 | ESS = 2,909 |  | ESS = 27,713 | ESS = 7,735 |  |
| Inpatient visits | | 0.05 (0.32) | 0.08 (0.38) | -0.03 (-0.05, -0.02)^b^ | 0.03 (0.24) | 0.07 (0.40) | -0.03 (-0.04, -0.02)^b^ |
| Inpatient days | | 0.11 (0.89) | 0.18 (1.11) | -0.07 (-0.11, -0.03)^b^ | 0.07 (0.69) | 0.14 (1.08) | -0.07 (-0.10, -0.05)^b^ |
| Telehealth visits | | 0.08 (0.96) | 0.04 (0.61) | 0.04 (0.01, 0.07)^b^ | 0.06 (0.73) | 0.05 (0.53) | 0.02 (0.00, 0.03)^b^ |
| **Outpatient visits** | |  |  |  |  |  |  |
|  | All | 4.13 (7.30) | 4.80 (7.67) | -0.67 (-0.98, -0.37)^b^ | 3.70 (6.51) | 4.51 (7.06) | -0.82 (-0.99, -0.64)^b^ |
|  | Clinic | 0.21 (2.44) | 0.22 (2.62) | -0.01 (-0.10, 0.08) | 0.16 (1.53) | 0.17 (1.62) | -0.01 (-0.04, 0.02) |
|  | Office | 2.92 (5.79) | 3.30 (5.88) | -0.38 (-0.63, -0.13)^b^ | 2.69 (5.44) | 3.22 (5.69) | -0.54 (-0.68, -0.40)^b^ |
|  | Hospital | 1.00 (2.74) | 1.28 (3.33) | -0.28 (-0.40, -0.16)^b^ | 0.85 (2.51) | 1.12 (2.86) | -0.27 (-0.34, -0.20)^b^ |
| Other/unknown visits | | 1.13 (3.19) | 1.31 (4.42) | -0.18 (-0.34, -0.02)^b^ | 0.74 (2.47) | 0.78 (3.25) | -0.04 (-0.11, 0.04) |
| Surgeries | | 0.01 (0.12) | 0.02 (0.15) | -0.01 (-0.01, -0.00)^b^ | 0.00 (0.07) | 0.01 (0.13) | -0.01 (-0.01, 0.00) |
| **Prescriptions** | |  |  |  |  |  |  |
|  | All | 18.84 (17.30) | 20.16 (18.57) | -1.32 (-2.05, -0.58)^b^ | 15.67 (14.90) | 17.03 (16.49) | -1.37 (-1.77, -0.96)^b^ |
|  | Obesity-specific | 1.62 (3.91) | 1.69 (4.14) | -0.06 (-0.23, 0.10) | 1.03 (3.11) | 0.87 (2.86) | 0.16 (0.09, 0.24)^b^ |
|  |  |  |  |  |  |  |  |
| **24 months post-index** | | ESS = 3,609 | ESS = 1,243 |  | ESS = 7,834 | ESS = 3,279 |  |
| Inpatient visits | | 0.12 (0.72) | 0.16 (0.71) | -0.03 (-0.07, 0.01) | 0.07 (0.38) | 0.12 (0.46) | -0.05 (-0.06, -0.03)^b^ |
| Inpatient days | | 0.28 (2.05) | 0.33 (1.59) | -0.04 (-0.15, 0.06) | 0.17 (1.21) | 0.27 (1.34) | -0.10 (-0.15, -0.05)^b^ |
| Telehealth visits | | 0.17 (1.64) | 0.14 (1.69) | 0.03 (-0.10, 0.16) | 0.13 (1.48) | 0.12 (1.29) | 0.01 (-0.04, 0.07)^b^ |
| **Outpatient visits** | |  |  |  |  |  |  |
|  | All | 8.71 (12.56) | 9.18 (12.62) | -0.48 (-1.30, 0.34) | 7.92 (11.40) | 8.54 (11.76) | -0.62 (-1.09, -0.14)^b^ |
|  | Clinic | 0.45 (4.06) | 0.45 (3.39) | 0.01 (-0.23, 0.24) | 0.30 (1.63) | 0.34 (2.10) | -0.05 (-0.11, 0.02) |
|  | Office | 6.15 (9.99) | 6.41 (10.15) | -0.26 (-0.94, 0.41) | 5.75 (9.38) | 5.99 (9.34) | -0.24 (-0.62, 0.14) |
|  | Hospital | 2.10 (4.79) | 2.32 (4.76) | -0.22 (-0.51, 0.07) | 1.87 (4.67) | 2.20 (4.66) | -0.33 (-0.53, -0.13)^b^ |
| Other/unknown visits | | 2.42 (5.62) | 2.31 (6.29) | 0.11 (-0.29, 0.51) | 1.53 (4.21) | 1.43 (4.95) | 0.10 (-0.08, 0.28) |
| Surgeries | | 0.02 (0.16) | 0.03 (0.20) | -0.01 (-0.02, 0.00) | 0.01 (0.12) | 0.02 (0.17) | -0.01 (-0.01, 0.00) |
| **Prescriptions** | |  |  |  |  |  |  |
|  | All | 40.32 (33.45) | 42.58 (34.71) | -2.26 (-4.52, 0.00) | 33.19 (28.79) | 36.62 (31.43) | -3.42 (-4.68, -2.17)^b^ |
|  | Obesity-specific | 3.50 (7.38) | 3.81 (8.29) | -0.31 (-0.83, 0.20) | 2.23 (6.04) | 1.96 (5.55) | 0.27 (0.03, 0.51)^b^ |

^a^ESS: Effective sample size

^b^Significant at a .05 level

**Table S5**. Healthcare resource utilization rates by service type according to Noom engagement level

| Service type | | Non-Noom users, Mean (SD) | Low-engaged Noom users, Mean (SD) | High-engaged Noom users, Mean (SD) | Low-engaged Noom users vs. non-Noom users, MD (95% CI) | High-engaged Noom users vs. non-Noom users, MD (95% CI) | High-engaged Noom vs. low-engaged Noom users, MD (95% CI) |
| --- | --- | --- | --- | --- | --- | --- | --- |
| **12 months post-index** | | ESS = 10,544 | ESS = 9,351 | ESS = 30,704 |  |  |  |
| Inpatient visits | | 0.07 (0.40) | 0.04 (0.24) | 0.04 (0.27) | -0.03 (-0.04, -0.02)^b^ | -0.03 (-0.04, -0.02)^b^ | 0.00 (-0.01, 0.01) |
| Inpatient days | | 0.15 (1.09) | 0.10 (0.99) | 0.08 (0.68) | -0.06 (-0.09, -0.02)^b^ | -0.08 (-0.10, -0.05)^b^ | -0.02 (-0.05, 0.01) |
| Telehealth visits | | 0.05 (0.56) | 0.06 (0.80) | 0.07 (0.82) | 0.02 (0.00, 0.04)^b^ | 0.02 (0.01, 0.04)^b^ | 0.01 (-0.02, 0.03) |
| **Outpatient visits** | |  |  |  |  |  |  |
|  | All | 4.61 (7.26) | 3.76 (6.64) | 3.86 (6.83) | -0.85 (-1.08, -0.62)^b^ | -0.74 (-0.93, -0.56)^b^ | 0.11 (-0.08, 0.29) |
|  | Clinic | 0.18 (1.99) | 0.18 (1.89) | 0.17 (1.87) | 0.00 (-0.06, 0.06) | -0.01 (-0.06, 0.03) | -0.01 (-0.06, 0.04) |
|  | Office | 3.25 (5.75) | 2.69 (5.38) | 2.79 (5.62) | -0.56 (-0.74, -0.37)^b^ | -0.46 (-0.62, -0.31)^b^ | 0.09 (-0.06, 0.24) |
|  | Hospital | 1.17 (3.02) | 0.88 (2.48) | 0.90 (2.61) | -0.29 (-0.38, -0.20)^b^ | -0.27 (-0.35, -0.19)^b^ | 0.02 (-0.05, 0.09) |
| Other/unknown visits | | 0.95 (3.67) | 0.82 (2.57) | 0.88 (2.79) | -0.13 (-0.23, -0.02)^b^ | -0.07 (-0.16, 0.02) | 0.06 (-0.02, 0.13) |
| Surgeries | | 0.01 (0.14) | 0.01 (0.10) | 0.01 (0.08) | -0.01 (-0.01, 0.00) | -0.01 (-0.01, -0.00)^b^ | 0.00 (0.00, 0.00) |
| **Prescriptions** | |  |  |  |  |  |  |
|  | All | 18.01 (17.23) | 17.37 (16.12) | 16.42 (15.62) | -0.64 (-1.20, -0.09)^b^ | -1.59 (-2.04, -1.15)^b^ | -0.95 (-1.40, -0.50)^b^ |
|  | Obesity-specific | 1.12 (3.34) | 1.34 (3.49) | 1.17 (3.35) | 0.21 (0.10, 0.32)^b^ | 0.05 (-0.04, 0.14) | -0.16 (-0.26, -0.07)^b^ |
|  |  |  |  |  |  |  |  |
| **24 months post-index** | | ESS = 4,484 | ESS = 2,749 | ESS = 8,678 |  |  |  |
| Inpatient visits | | 0.13 (0.55) | 0.10 (0.50) | 0.09 (0.53) | -0.03 (-0.06, -0.00)^b^ | -0.04 (-0.07, -0.02)^b^ | -0.01 (-0.04, 0.01) |
| Inpatient days | | 0.29 (1.43) | 0.23 (1.49) | 0.20 (1.52) | -0.06 (-0.14, 0.01) | -0.09 (-0.15, -0.03)^b^ | -0.03 (-0.10, 0.04) |
| Telehealth visits | | 0.13 (1.43) | 0.11 (1.11) | 0.16 (1.63) | -0.02 (-0.09, 0.05) | 0.03 (-0.04, 0.10) | 0.05 (-0.02, 0.11) |
| **Outpatient visits** | |  |  |  |  |  |  |
|  | All | 8.74 (12.04) | 8.01 (11.72) | 8.22 (11.84) | -0.73 (-1.40, -0.07)^b^ | -0.52 (-1.04, -0.00)^b^ | 0.21 (-0.38, 0.81) |
|  | Clinic | 0.37 (2.57) | 0.38 (2.88) | 0.34 (2.59) | 0.00 (-0.14, 0.14) | -0.04 (-0.15, 0.08) | -0.04 (-0.19, 0.11) |
|  | Office | 6.12 (9.60) | 5.69 (9.14) | 5.94 (9.72) | -0.44 (-0.97, 0.09) | -0.18 (-0.60, 0.24) | 0.26 (-0.21, 0.72) |
|  | Hospital | 2.24 (4.70) | 1.95 (4.54) | 1.94 (4.74) | -0.30 (-0.56, -0.03)^b^ | -0.30 (-0.50, -0.10)^b^ | -0.01 (-0.24, 0.23) |
| Other/unknown visits | | 1.71 (5.42) | 1.85 (4.78) | 1.80 (4.70) | 0.14 (-0.14, 0.41) | 0.09 (-0.13, 0.31) | -0.05 (-0.29, 0.19) |
| Surgeries | | 0.02 (0.18) | 0.02 (0.14) | 0.02 (0.14) | -0.01 (-0.01, 0.00) | -0.01 (-0.02, -0.00)^b^ | 0.00 (-0.01, 0.00) |
| **Prescriptions** | |  |  |  |  |  |  |
|  | All | 38.49 (32.62) | 37.54 (32.04) | 34.75 (30.03) | -0.95 (-2.78, 0.87) | -3.74 (-5.13, -2.35)^b^ | -2.79 (-4.41, -1.17)^b^ |
|  | Obesity-specific | 2.54 (6.59) | 3.03 (6.95) | 2.51 (6.36) | 0.49 (0.10, 0.87)^b^ | -0.04 (-0.32, 0.25) | -0.52 (-0.86, -0.18)^b^ |

^a^ESS: Effective sample size

^b^Significant at a .05 level

**Table S6**. Healthcare resource utilization rates by service type according to Noom use duration

| Service type | | Non-Noom use, mean (SD) | Noom use < 6 months, mean (SD) | Noom use ≥ 6 months, mean (SD) | Noom use < 6 months vs. non-Noom use, MD (95% CI) | Noom use ≥ 6 months vs. non-Noom use, MD (95% CI) | Noom use ≥ 6 months vs. Noom use < 6 months, MD (95% CI) |
| --- | --- | --- | --- | --- | --- | --- | --- |
| **12 months post-index** | | ESS = 10,544 | ESS = 14,402 | ESS = 25,391 |  |  |  |
| Inpatient visits | | 0.07 (0.40) | 0.04 (0.30) | 0.04 (0.25) | -0.03 (-0.04, -0.02)^b^ | -0.03 (-0.04, -0.02)^b^ | 0.00 (-0.01, 0.00) |
| Inpatient days | | 0.15 (1.09) | 0.09 (0.97) | 0.08 (0.65) | -0.06 (-0.09, -0.03)^b^ | -0.08 (-0.10, -0.05)^b^ | -0.02 (-0.04, 0.01) |
| Telehealth visits | | 0.05 (0.55) | 0.06 (0.66) | 0.07 (0.87) | 0.02 (-0.00, 0.03) | 0.03 (0.01, 0.04)^b^ | 0.01 (-0.01, 0.03) |
| **Outpatient visits** | |  |  |  |  |  |  |
|  | All | 4.61 (7.26) | 3.84 (6.92) | 3.83 (6.72) | -0.76 (-0.98, -0.55)^b^ | -0.77 (-0.97, -0.58)^b^ | -0.01 (-0.18, 0.16) |
|  | Clinic | 0.18 (2.00) | 0.19 (2.24) | 0.17 (1.74) | 0.01 (-0.06, 0.07) | -0.01 (-0.06, 0.03) | -0.02 (-0.08, 0.04) |
|  | Office | 3.25 (5.75) | 2.75 (5.59) | 2.77 (5.54) | -0.50 (-0.67, -0.32)^b^ | -0.48 (-0.64, -0.33)^b^ | 0.01 (-0.12, 0.15) |
|  | Hospital | 1.17 (3.02) | 0.90 (2.57) | 0.90 (2.57) | -0.27 (-0.36, -0.19)^b^ | -0.28 (-0.35, -0.20)^b^ | 0.00 (-0.07, 0.06) |
| Other/unknown visits | | 0.95 (3.67) | 0.86 (2.60) | 0.87 (2.79) | -0.09 (-0.19, 0.01) | -0.08 (-0.17, 0.01) | 0.01 (-0.05, 0.08) |
| Surgeries | | 0.01 (0.14) | 0.01 (0.09) | 0.01 (0.08) | -0.01 (-0.01, -0.00)^b^ | -0.01 (-0.01, 0.00)^b^ | 0.00 (-0.00, 0.00) |
| **Prescriptions** | |  |  |  |  |  |  |
|  | All | 18.02 (17.23) | 17.08 (16.25) | 16.44 (15.51) | -0.94 (-1.45, -0.43)^b^ | -1.58 (-2.03, -1.12)^b^ | -0.64 (-1.04, -0.24)^b^ |
|  | Obesity-specific | 1.12 (3.34) | 1.24 (3.39) | 1.20 (3.39) | 0.12 (0.01, 0.22)^b^ | 0.08 (-0.02, 0.17) | -0.04 (-0.12, 0.04) |
|  |  |  |  |  |  |  |  |
| **24 months post-index** | | ESS = 4,482 | ESS = 3,941 | ESS = 7,397 |  |  |  |
| Inpatient visits | | 0.13 (0.55) | 0.10 (0.62) | 0.08 (0.45) | -0.03 (-0.06, -0.00)^b^ | -0.05 (-0.07, -0.03)^b^ | -0.02 (-0.04, 0.01) |
| Inpatient days | | 0.29 (1.43) | 0.21 (1.37) | 0.20 (1.61) | -0.07 (-0.14, -0.01)^b^ | -0.08 (-0.14, -0.02)^b^ | -0.01 (-0.08, 0.06) |
| Telehealth visits | | 0.13 (1.42) | 0.17 (1.88) | 0.13 (1.29) | 0.04 (-0.05, 0.13) | 0.01 (-0.06, 0.07) | -0.03 (-0.11, 0.05) |
| **Outpatient visits** | |  |  |  |  |  |  |
|  | All | 8.74 (12.04) | 8.28 (12.27) | 8.13 (11.58) | -0.46 (-1.09, 0.18) | -0.62 (-1.14, -0.09)^b^ | -0.16 (-0.72, 0.40) |
|  | Clinic | 0.38 (2.58) | 0.42 (3.93) | 0.31 (1.79) | 0.04 (-0.18, 0.27) | -0.06 (-0.15, 0.02) | -0.10 (-0.32, 0.11) |
|  | Office | 6.12 (9.60) | 5.91 (9.76) | 5.87 (9.52) | -0.21 (-0.71, 0.28) | -0.25 (-0.68, 0.18) | -0.04 (-0.47, 0.40) |
|  | Hospital | 2.24 (4.69) | 1.96 (4.74) | 1.94 (4.67) | -0.28 (-0.53, -0.04)^b^ | -0.30 (-0.51, -0.10)^b^ | -0.02 (-0.24, 0.20) |
| Other/unknown visits | | 1.71 (5.43) | 1.86 (4.81) | 1.78 (4.68) | 0.15 (-0.13, 0.44) | 0.07 (-0.15, 0.29) | -0.08 (-0.33, 0.17) |
| Surgeries | | 0.02 (0.18) | 0.02 (0.14) | 0.02 (0.13) | -0.01 (-0.01, 0.00) | -0.01 (-0.02, 0.00)^b^ | 0.00 (-0.01, 0.00) |
| **Prescriptions** | |  |  |  |  |  |  |
|  | All | 38.50 (32.63) | 36.20 (31.24) | 35.04 (30.13) | -2.30 (-3.98, -0.63)^b^ | -3.46 (-4.89, -2.04)^b^ | -1.16 (-2.63, 0.31) |
|  | Obesity-specific | 2.54 (6.59) | 2.67 (6.51) | 2.60 (6.49) | 0.13 (-0.21, 0.47) | 0.05 (-0.24, 0.35) | -0.07 (-0.37, 0.22) |

^a^ESS: Effective sample size

^b^Significant at a .05 level

**Table S7**. Healthcare costs by service type among patients with at least 1 encounter

| Service type | | Noom users | Non-Noom users |  | | |
| --- | --- | --- | --- | --- | --- | --- |
|  |  | Mean (SD) | Mean (SD) | Mean difference (95% CI) | P value |  |
| **12 months post-index^a^** | | | | | |  |
| Inpatient services | | $820.77 (1,994.56) | $819.19 (1,994.21) | 1.59 (-192.54, 195.71) | 0.987 |  |
| Telehealth services | | $236.23 (477.76) | $175.01 (278.82) | 61.23 (14.55, 107.90) | 0.010^b^ |  |
| **Outpatient services** | |  |  |  |  |  |
|  | All | $706.71 (1,582.38) | $801.52 (1,991.97) | -94.81 (-140.57, -49.05) | <0.001^b^ |  |
|  | Clinic | $178.93 (414.10) | $209.33 (506.86) | -30.40 (-58.75, -2.05) | 0.036^b^ |  |
|  | Office | $471.97 (1,064.48) | $550.99 (1,428.44) | -79.02 (-114.46, -43.58) | <0.001^b^ |  |
|  | Hospital | $622.89 (1,614.70) | $619.39 (1,810.42) | 3.50 (-56.76, 63.77) | 0.909 |  |
| Other/unknown services | | $169.00 (2,100.37) | $143.47 (431.72) | 25.52 (-17.25, 68.29) | 0.242 |  |
| **Prescriptions** | |  |  |  |  |  |
|  | All | $3,042.22 (10,446.88) | $3,380.75 (13,882.28) | -338.52 (-605.57, -71.47) | 0.013^b^ |  |
|  | Obesity-specific | $2,292.18 (6,597.84) | $2,665.34 (6,867.72) | -373.16 (-719.20, -27.12) | 0.035^b^ |  |
|  | |  |  |  |  |  |
| **24 months post-index^a^** | | | | | |  |
| Inpatient services | | $863.19 (2,160.53) | $691.64 (1,266.84) | 171.55 (-17.04, 360.15) | 0.075 |  |
| Telehealth services | | $335.09 (965.40) | $221.95 (414.77) | 113.14 (5.75, 220.53) | 0.039^b^ |  |
| **Outpatient services** | |  |  |  |  |  |
|  | All | $1,163.06 (2,276.39) | $1,217.60 (2,149.74) | -54.54 (-133.20, 24.12) | 0.174 |  |
|  | Clinic | $241.28 (587.19) | $280.46 (785.47) | -39.18 (-93.71, 15.35) | 0.159 |  |
|  | Office | $783.21 (1,645.39) | $800.07 (1,567.95) | -16.87 (-76.22, 42.49) | 0.578 |  |
|  | Hospital | $781.61 (1,803.43) | $790.01 (1,585.80) | -8.40 (-87.13, 70.32) | 0.834 |  |
| Other/unknown services | | $201.90 (722.07) | $196.00 (791.49) | 5.90 (-23.59, 35.39) | 0.695 |  |
| **Prescriptions** | |  |  |  |  |  |
|  | All | $6,352.10 (19,630.54) | $7,516.68 (29,262.89) | -1,164.58 (-2,014.79, -314.37) | 0.007^b^ |  |
|  | Obesity-specific | $3,614.68 (10,620.92) | $4,728.05 (12,907.71) | -1,113.36 (-1,996.42, -230.31) | 0.013^b^ |  |

^a^Sample sizes differ by service type.

^b^Significant at a .05 level

**Table S8**. Healthcare costs by service type according to type 2 diabetes status

| Service type | | Diabetic | | | Non-diabetic | | |
| --- | --- | --- | --- | --- | --- | --- | --- |
|  |  | Noom users, Mean (SD) | Non-Noom users, Mean (SD) | Mean difference (95% CI) | Noom users, Mean (SD) | Non-Noom users, Mean (SD) | Mean difference, (95% CI) |
| **12 months post-index** | | ESS = 2,200 | ESS = 992 |  | ESS = 38,154 | ESS = 9,570 |  |
| Inpatient services | | $59.15 (486.94) | $69.78 (485.72) | -10.63 (-46.35, 25.08) | $21.98 (359.06) | $41.95 (498.73) | -19.98 (-30.45, -9.51)^b^ |
| Telehealth services | | $7.30 (98.45) | $3.92 (56.94) | 3.38 (-2.05, 8.82) | $6.00 (84.37) | $3.48 (45.40) | 2.52 (1.30, 3.75)^b^ |
| **Outpatient services** | |  |  |  |  |  |  |
|  | All | $771.52 (1,762.96) | $890.37 (2,287.98) | -118.85 (-289.62, 51.93) | $474.86 (1,328.83) | $591.82 (1,723.75) | -116.96 (-152.45, -81.47)^b^ |
|  | Clinic | $24.57 (180.49) | $24.34 (126.09) | 0.23 (-10.56, 11.02) | $15.49 (130.54) | $15.51 (153.93) | -0.02 (-2.61, 2.57) |
|  | Office | $408.94 (1,069.82) | $493.20 (1,805.39) | -84.26 (-212.12, 43.61) | $259.44 (818.15) | $335.56 (1,089.33) | -76.12 (-98.16, -54.08)^b^ |
|  | Hospital | $338.01 (1,312.86) | $372.83 (1,169.56) | -34.82 (-135.21, 65.58) | $199.93 (952.63) | $240.75 (1,195.32) | -40.82 (-66.12, -15.52)^b^ |
| Other/unknown services | | $76.70 (403.00) | $84.60 (403.44) | -7.89 (-38.16, 22.37) | $45.77 (1,147.62) | $38.46 (222.67) | 7.31 (-5.51, 20.14) |
| **Prescriptions** | |  |  |  |  |  |  |
|  | All | $8,345.87 (16,313.18) | $7,835.45 (14,636.37) | 510.42 (-601.58, 1,622.42) | $2,516.83 (9,535.39) | $2,751.36 (13,290.90) | -234.53 (-490.70, 21.64) |
|  | Obesity-specific | $2,920.65 (7,562.69) | $2,675.83 (7,359.93) | 244.82 (-317.46, 807.11) | $273.36 (2,349.18) | $264.97 (2,167.75) | 8.39 (-42.11, 58.90) |
| Overall services | | $9,260.55 (16,510.51) | $8,884.12 (14,855.53) | 376.43 (-752.37, 1,505.23) | $3,065.44 (9,769.39) | $3,427.07 (13,455.37) | -361.63 (-621.95, -101.31)^b^ |
|  | |  |  |  |  |  |  |
| **24 months post-index** | | ESS = 495 | ESS = 386 |  | ESS = 10,972 | ESS = 4,100 |  |
| Inpatient services | | 113.48 (639.13) | $87.69 (400.62) | 25.79 (-36.52, 88.09) | $51.33 (578.19) | $60.35 (430.53) | -9.02 (-25.45, 7.42) |
| Telehealth services | | 9.03 (61.68) | $7.00 (71.55) | 2.03 (-7.25, 11.31) | $14.32 (213.82) | $9.25 (96.29) | 5.07 (-0.01, 10.15) |
| **Outpatient services** | |  |  |  |  |  |  |
|  | All | 1,636.48 (4,200.18) | $1,295.36 (1,612.17) | 341.11 (8.71, 673.52)^b^ | $963.82 (1,964.54) | $1,060.94 (2,096.13) | -97.12 (-170.00, -24.23)^b^ |
|  | Clinic | 32.39 (158.87) | $50.52 (258.17) | -18.12 (-48.68, 12.43) | $35.25 (243.30) | $37.74 (311.73) | -2.49 (-11.03, 6.06) |
|  | Office | 981.99 (3,598.69) | $716.87 (900.25) | 265.12 (4.65, 525.58)^b^ | $549.21 (1,215.42) | $587.82 (1,436.09) | -38.61 (-85.33, 8.11) |
|  | Hospital | 622.09 (1,858.64) | $527.97 (1,097.55) | 94.12 (-79.08, 267.32) | $379.36 (1,299.12) | $435.38 (1,263.27) | -56.02 (-102.91, -9.14)^b^ |
| Other/unknown services | | $173.89 (528.82) | $156.00 (1,513.61) | 17.89 (-74.84, 110.62) | $79.38 (473.84) | $73.55 (287.85) | 5.83 (-5.17, 16.84) |
| **Prescriptions** | |  |  |  |  |  |  |
|  | All | $17,299.62 (30,367.93) | $16,823.75 (27,651.23) | 475.87 (-3,239.99, 4,191.72) | $5,589.08 (18,436.38) | $6,498.55 (28,931.87) | -909.46 (-1,759.23, -59.70)^b^ |
|  | Obesity-specific | $6,444.57 (15,578.87) | $5,911.56 (14,568.35) | 533.01 (-1,452.69, 2,518.70) | $605.96 (4,167.52) | $719.39 (5,220.67) | -113.43 (-300.70, 73.83) |
| Overall services | | $19,232.49 (30,838.24) | $18,369.81 (27,899.97) | 862.68 (-2,893.04, 4,618.41) | $6,697.94 (18,708.61) | $7,702.63 (29,144.53) | -1,004.69 (-1,864.21, -145.17)^b^ |

^a^ESS: Effective sample size

^b^Significant at a .05 level **Table S9**. Healthcare costs by service type according to hypertension status

| Service type | | Hypertensive | | | Non-hypertensive | | |
| --- | --- | --- | --- | --- | --- | --- | --- |
|  |  | Noom users, Mean (SD) | Non-Noom users, Mean (SD) | Mean difference (95% CI) | Noom users, Mean (SD) | Non-Noom users, Mean (SD) | Mean difference, (95% CI) |
| **12 months post-index** | | ESS = 7,256 | ESS = 2,594 |  | ESS = 33,119 | ESS = 7,981 |  |
| Inpatient services | | $43.99 (517.48) | $61.21 (482.74) | -17.22 (-37.75, 3.30) | $19.48 (322.28) | $39.39 (501.86) | -19.91 (-31.43, -8.40)^b^ |
| Telehealth services | | $5.80 (61.53) | $4.05 (44.76) | 1.75 (-0.47, 3.97) | $6.15 (90.00) | $3.36 (46.96) | 2.79 (1.39, 4.18)^b^ |
| **Outpatient services** | |  |  |  |  |  |  |
|  | All | $722.76 (1,998.06) | $836.70 (2,540.69) | -113.94 (-223.55, -4.33)^b^ | $437.77 (1,151.49) | $553.28 (1,483.16) | -115.51 (-148.72, -82.30)^b^ |
|  | Clinic | $20.55 (210.01) | $19.03 (253.67) | 1.52 (-6.77, 9.80) | $14.95 (108.40) | $15.45 (105.40) | -0.49 (-2.76, 1.78) |
|  | Office | $386.95 (1,438.81) | $450.68 (1,321.36) | -63.73 (-123.56, -3.90)^b^ | $240.14 (607.31) | $319.31 (1,116.84) | -79.17 (-103.27, -55.07)^b^ |
|  | Hospital | $315.27 (1,284.79) | $366.99 (2,031.14) | -51.73 (-138.21, 34.75) | $182.68 (888.19) | $218.52 (799.85) | -35.84 (-55.47, -16.22)^b^ |
| Other/unknown services | | 50.69 (328.75) | $54.26 (274.37) | -3.57 (-16.17, 9.02) | $46.88 (1,232.66) | $38.87 (233.52) | 8.01 (-6.88, 22.89) |
| **Prescriptions** | |  |  |  |  |  |  |
|  | All | $4,080.12 (12,003.93) | $3,883.80 (11,083.01) | 196.32 (-314.85, 707.48) | $2,574.36 (9,648.62) | $2,973.15 (14,093.49) | -398.79 (-689.47, -108.11)^b^ |
|  | Obesity-specific | $798.16 (4,073.18) | $771.49 (4,270.54) | 26.67 (-162.53, 215.87) | $343.50 (2,671.19) | $378.95 (2,582.06) | -35.45 (-100.02, 29.12) |
| Overall services | | $4,903.36 (12,298.30) | $4,840.03 (11,449.42) | 63.33 (-462.72, 589.38) | $3,084.64 (9,860.82) | $3,608.06 (14,225.63) | -523.42 (-817.98, -228.85)^b^ |
|  | |  |  |  |  |  |  |
| **24 months post-index** | | ESS = 1,576 | ESS = 991 |  | ESS = 9,904 | ESS = 3,495 |  |
| Inpatient services | | $72.59 (342.99) | $76.25 (456.81) | -3.66 (-33.49, 26.17) | $51.28 (616.35) | $58.84 (419.85) | -7.55 (-25.56, 10.45) |
| Telehealth services | | $8.43 (109.95) | $7.80 (93.85) | 0.63 (-7.01, 8.27) | $15.09 (222.21) | $9.41 (94.67) | 5.68 (0.14, 11.22)^b^ |
| **Outpatient services** | |  |  |  |  |  |  |
|  | All | $1,452.11 (3,029.77) | $1,354.16 (2,340.76) | 97.95 (-89.38, 285.28) | $914.71 (1,927.39) | $1,004.48 (1,970.32) | -89.77 (-165.42, -14.12)^b^ |
|  | Clinic | $37.75 (184.07) | $37.57 (416.59) | 0.18 (-20.05, 20.41) | $34.60 (248.59) | $39.14 (269.82) | -4.54 (-13.34, 4.26) |
|  | Office | $832.92 (2,296.19) | $735.55 (1,407.38) | 97.37 (-24.34, 219.08) | $523.34 (1,219.65) | $560.54 (1,395.56) | -37.19 (-87.15, 12.76) |
|  | Hospital | $581.44 (1,767.29) | $581.05 (1,571.49) | 0.40 (-123.32, 124.12) | $356.77 (1,235.09) | $404.81 (1,143.04) | -48.04 (-95.10, -0.98)^b^ |
| Other/unknown services | | $129.85 (920.47) | $92.54 (299.34) | 37.31 (-2.21, 76.84) | $75.89 (333.22) | $77.02 (561.30) | -1.12 (-14.52, 12.28) |
| **Prescriptions** | |  |  |  |  |  |  |
|  | All | $8,153.04 (19,900.91) | $8,578.62 (20,862.20) | -425.57 (-2,038.05, 1,186.90) | $5,850.63 (19,330.78) | $7,015.32 (30,830.92) | -1,164.69 (-2,133.16, -196.22)^b^ |
|  | Obesity-specific | $1,746.61 (8,181.25) | $1,813.92 (8,745.13) | -67.32 (-739.82, 605.18) | $762.25 (4,923.15) | $965.80 (5,969.79) | -203.55 (-437.43, 30.33) |
| Overall services | | $9,816.03 (20,427.93) | $10,109.37 (21,144.19) | -293.34 (-1,931.14, 1,344.46) | $6,907.61 (19,585.25) | $8,165.06 (31,041.87) | -1,257.45 (-2,236.68, -278.23)^b^ |

^a^ESS: Effective sample size

^b^Significant at a .05 level **Table S10**. Healthcare costs by service type according to index BMI

| Service type | | Index BMI ≥ 35 | | | Index BMI < 35 | | |
| --- | --- | --- | --- | --- | --- | --- | --- |
|  |  | Noom users, Mean (SD) | Non-Noom users, Mean (SD) | Mean difference (95% CI) | Noom users, Mean (SD) | Non-Noom users, Mean (SD) | Mean difference (95% CI) |
| **12 months post-index** | | ESS = 12,643 | ESS = 2,909 |  | ESS = 27,713 | ESS = 7,735 |  |
| Inpatient services | | $31.21 (338.69) | $49.25 (411.31) | -18.04 (-33.77, -2.30)^b^ | $21.14 (379.98) | $42.00 (532.82) | -20.86 (-33.51, -8.21)^b^ |
| Telehealth services | | $6.80 (94.50) | $2.96 (37.65) | 3.84 (1.65, 6.03)^b^ | $5.77 (80.94) | $3.77 (50.02) | 2.00 (0.57, 3.43)^b^ |
| **Outpatient services** | |  |  |  |  |  |  |
|  | All | $534.18 (1,627.64) | $646.78 (1,617.91) | -112.60 (-177.25, -47.95)^b^ | $474.43 (1,226.05) | $603.03 (1,849.49) | -128.59 (-170.99, -86.20)^b^ |
|  | Clinic | $17.46 (127.42) | $16.44 (97.75) | 1.02 (-2.83, 4.86) | $15.41 (136.81) | $16.16 (171.06) | -0.75 (-3.97, 2.47) |
|  | Office | $293.07 (1,202.12) | $358.96 (1,231.64) | -65.89 (-115.48, -16.30)^b^ | $257.61 (612.37) | $344.07 (1,136.11) | -86.46 (-110.81, -62.12)^b^ |
|  | Hospital | $223.65 (995.48) | $271.38 (918.99) | -47.73 (-84.48, -10.99)^b^ | $201.42 (970.69) | $242.80 (1,300.87) | -41.38 (-73.01, -9.75)^b^ |
| Other/unknown services | | $71.43 (1,791.13) | $55.45 (240.83) | 15.98 (-16.42, 48.37) | $37.28 (631.32) | $36.27 (244.28) | 1.01 (-9.52, 11.55) |
| **Prescriptions** | |  |  |  |  |  |  |
|  | All | $3,515.45 (11,227.44) | $3,856.29 (11,197.30) | -340.83 (-784.56, 102.89) | $2,580.89 (9,648.10) | $2,864.44 (14,403.44) | -283.54 (-588.82, 21.74) |
|  | Obesity-specific | $719.29 (3,915.65) | $746.05 (3,686.98) | -26.75 (-177.96, 124.45) | $305.75 (2,485.73) | $338.35 (2,692.82) | -32.60 (-97.52, 32.32) |
| Overall services | | $4,159.07 (11,580.89) | $4,610.73 (11,375.36) | -451.66 (-904.10, 0.78) | $3,119.52 (9,824.66) | $3,549.51 (14,583.62) | -429.98 (-740.08, -119.88)^b^ |
|  | |  |  |  |  |  |  |
| **24 months post-index** | | ESS = 3,609 | ESS = 1,243 |  | ESS = 7,834 | ESS = 3,279 |  |
| Inpatient services | | $66.23 (504.58) | $66.12 (400.88) | 0.11 (-23.76, 23.98) | $49.56 (612.60) | $61.00 (440.21) | -11.44 (-31.59, 8.71) |
| Telehealth services | | $17.70 (264.63) | $10.36 (102.93) | 7.34 (-3.55, 18.23) | $12.42 (178.30) | $8.46 (90.34) | 3.96 (-1.00, 8.91) |
| **Outpatient services** | |  |  |  |  |  |  |
|  | All | $1,083.42 (2,471.33) | $1,111.25 (1,947.63) | -27.82 (-153.66, 98.01) | $962.95 (1,989.45) | $1,066.15 (2,111.62) | -103.20 (-188.38, -18.01)^b^ |
|  | Clinic | $43.19 (331.81) | $44.30 (359.51) | -1.11 (-20.59, 18.37) | $31.54 (184.75) | $36.26 (280.56) | -4.73 (-12.95, 3.50) |
|  | Office | $606.00 (1,806.17) | $622.65 (1,257.24) | -16.65 (-100.98, 67.68) | $557.53 (1,259.57) | $587.40 (1,460.94) | -29.87 (-84.36, 24.61) |
|  | Hospital | $434.23 (1,459.16) | $444.29 (1,217.75) | -10.06 (-88.73, 68.61) | $373.89 (1,277.65) | $442.48 (1,265.53) | -68.60 (-123.33, -13.87)^b^ |
| Other/unknown services | | $113.24 (673.91) | $109.93 (360.71) | 3.32 (-22.89, 29.52)^b^ | $71.75 (357.50) | $66.77 (572.96) | 4.98 (-8.98, 18.93) |
| **Prescriptions** | |  |  |  |  |  |  |
|  | All | $7,778.10 (21,872.82) | $8,455.62 (20,702.63) | -677.52 (-2,066.44, 711.40) | $5,526.94 (18,224.53) | $6,847.30 (32,054.24) | -1,320.37 (-2,354.75, -285.98)^b^ |
|  | Obesity-specific | $1,515.76 (7,500.38) | $1,803.52 (7,821.12) | -287.76 (-800.06, 224.54) | $654.92 (4,453.29) | $848.70 (6,059.00) | -193.78 (-427.78, 40.22) |
| Overall services | | $9,058.70 (22,196.96) | $9,753.28 (21,059.64) | -694.58 (-2,108.11, 718.95) | $6,623.61 (18,521.25) | $8,049.68 (32,241.61) | -1,426.07 (-2,470.17, -381.97)^b^ |

^a^ESS: Effective sample size

^b^Significant at a .05 level

**Table S11**. Healthcare costs by service type according to Noom engagement level

| Service type | | Non-Noom users, mean (SD) | Low-engaged Noom users, mean (SD) | High-engaged Noom users, mean (SD) | Low-engaged Noom users vs. non-Noom users, MD (95% CI) | High-engaged Noom users vs. non-Noom users, MD (95% CI) | High-engaged Noom vs. low-engaged Noom users, MD (95% CI) |
| --- | --- | --- | --- | --- | --- | --- | --- |
| **12 months post-index** | | ESS = 10,544 | ESS =9,351 | ESS = 30,704 |  |  |  |
| Inpatient services | | $44.29 (497.39) | $22.69 (265.95) | $24.59 (391.29) | -21.60 (-34.95, -8.25)^b^ | -19.70 (-31.83, -7.57)^b^ | 1.90 (-6.98, 10.77) |
| Telehealth services | | $3.52 (46.48) | $5.59 (87.33) | $6.28 (84.68) | 2.07 (-0.16, 4.30) | 2.76 (1.23, 4.29)^b^ | 0.69 (-1.56, 2.94) |
| **Outpatient services** | |  |  |  |  |  |  |
|  | All | $616.71 (1,778.90) | $487.15 (1,286.96) | $495.44 (1,394.43) | -129.55 (-179.49, -79.61)^b^ | -121.27 (-164.47, -78.07)^b^ | 8.29 (-27.58, 44.15) |
|  | Clinic | $16.25 (151.60) | $16.31 (99.50) | $15.97 (142.35) | 0.05 (-3.48, 3.58) | -0.28 (-3.47, 2.91) | -0.34 (-3.44, 2.77) |
|  | Office | $348.72 (1,166.20) | $260.69 (708.38) | $272.01 (890.54) | -88.03 (-119.00, -57.05)^b^ | -76.71 (-104.62, -48.80)^b^ | 11.32 (-9.81, 32.44) |
|  | Hospital | $251.73 (1,193.39) | $210.15 (987.54) | $207.46 (975.46) | -41.58 (-77.28, -5.88)^b^ | -44.28 (-74.13, -14.43)^b^ | -2.70 (-29.28, 23.89) |
| Other/unknown services | | $42.32 (243.34) | $62.92 (1,895.05) | $41.93 (609.27) | 20.59 (-20.79, 61.97) | -0.39 (-10.99, 10.21) | -20.98 (-62.76, 20.80) |
| **Prescriptions** | |  |  |  |  |  |  |
|  | All | $3,174.73 (13,464.32) | $2,913.93 (9,457.02) | $2,851.33 (10,346.29) | -260.80 (-623.60, 101.99) | -323.41 (-631.97, -14.84)^b^ | -62.60 (-344.36, 219.15) |
|  | Obesity-specific | $466.64 (3,046.60) | $477.95 (3,016.47) | $415.62 (2,991.73) | 11.30 (-88.29, 110.89) | -51.02 (-131.09, 29.05) | -62.33 (-144.22, 19.57) |
| Overall services | | $3,881.57 (13,644.05) | $3,492.28 (9,821.63) | $3,419.57 (10,542.06) | -389.29 (-760.31, -18.27)^b^ | -462.00 (-775.62, -148.39)^b^ | -72.71 (-362.07, 216.65) |
|  | |  |  |  |  |  |  |
| **24 months post-index** | | ESS = 4,484 | ESS = 2,749 | ESS = 8,678 |  |  |  |
| Inpatient services | | $62.63 (428.36) | $63.58 (656.42) | $51.73 (554.42) | 0.94 (-29.34, 31.22) | -10.90 (-30.46, 8.66) | -11.84 (-41.48, 17.79) |
| Telehealth services | | $9.06 (94.51) | $8.71 (102.87) | $15.61 (231.10) | -0.35 (-5.66, 4.95) | 6.54 (-0.25, 13.34) | 6.90 (-0.22, 14.01)^b^ |
| **Outpatient services** | |  |  |  |  |  |  |
|  | All | $1,080.28 (2,060.56) | $996.49 (2,048.10) | $1,002.11 (2,191.52) | -83.78 (-194.40, 26.83) | -78.16 (-166.70, 10.37) | 5.62 (-93.23, 104.46) |
|  | Clinic | $38.79 (307.78) | $39.59 (279.61) | $34.38 (233.12) | 0.80 (-12.95, 14.55) | -4.41 (-15.31, 6.49) | -5.21 (-18.89, 8.47) |
|  | Office | $598.44 (1,399.25) | $561.83 (1,469.87) | $576.51 (1,459.69) | -36.61 (-113.57, 40.35) | -21.93 (-79.02, 35.17) | 14.68 (-55.14, 84.51) |
|  | Hospital | $443.05 (1,250.23) | $395.07 (1,152.64) | $391.22 (1,385.60) | -47.98 (-113.35, 17.40) | -51.83 (-108.35, 4.70) | -3.85 (-60.33, 52.63) |
| Other/unknown services | | $80.33 (515.60) | $90.34 (375.55) | $83.25 (513.01) | 10.01 (-10.83, 30.85) | 2.92 (-13.37, 19.21) | -7.09 (-27.48, 13.30) |
| **Prescriptions** | |  |  |  |  |  |  |
|  | All | $7,349.55 (28,937.10) | $6,949.48 (21,029.93) | $5,982.97 (18,962.62) | -400.07 (-1,761.96, 961.82) | -1,366.57 (-2,377.74, -355.40)^b^ | -966.50 (-2,105.49, 172.49) |
|  | Obesity-specific | $1,148.90 (6,675.98) | $1,132.29 (6,317.73) | $842.15 (5,252.65) | -16.62 (-396.75, 363.51) | -306.75 (-579.96, -33.54)^b^ | -290.14 (-615.02, 34.75) |
| Overall services | | $8,581.85 (29,161.04) | $8,108.60 (21,293.76) | $7,135.68 (19,294.43) | -473.26 (-1,849.31, 902.80) | -1,446.18 (-2,469.70, -422.66)^b^ | -972.92 (-2,124.68, 178.84) |

^a^ESS: Effective sample size

^b^Significant at a .05 level

**Table S12**. Healthcare costs by service type according to Noom use duration

| Service type | | Non-Noom users*, Mean (SD) | Noom use < 6m*, Mean (SD) | Noom use ≥ 6m*, Mean (SD) | Noom use < 6m vs. non-Noom users, MD (95% CI) | Noom use ≥ 6m vs. non-Noom users, MD (95% CI) | Noom use ≥ 6m vs. Noom use < 6m, MD (95% CI) |
| --- | --- | --- | --- | --- | --- | --- | --- |
| **12 months post-index** | | ESS = 10,544 | ESS = 14,402 | ESS = 25,391 |  |  |  |
| Inpatient services | | $44.24 (497.55) | $24.61 (400.08) | $23.98 (342.76) | -19.63 (-33.20, -6.06)^b^ | -20.26 (-32.33, -8.19)^b^ | -0.63 (-9.75, 8.49) |
| Telehealth services | | $3.52 (46.49) | $5.75 (76.49) | $6.20 (89.10) | 2.23 (0.50, 3.97)^b^ | 2.68 (1.02, 4.35)^b^ | 0.45 (-1.44, 2.33) |
| **Outpatient services** | |  |  |  |  |  |  |
|  | All | $616.76 (1,781.29) | $496.91 (1,551.54) | $490.30 (1,295.60) | -119.85 (-170.31, -69.38)^b^ | -126.46 (-169.88, -83.04)^b^ | -6.61 (-43.19, 29.97) |
|  | Clinic | $16.25 (151.69) | $16.52 (113.32) | $15.90 (148.21) | 0.27 (-3.32, 3.85) | -0.36 (-3.71, 3.00) | -0.63 (-3.96, 2.70) |
|  | Office | $348.72 (1,165.62) | $275.32 (1,212.41) | $265.58 (658.15) | -73.40 (-109.55, -37.25)^b^ | -83.14 (-110.10, -56.18)^b^ | -9.74 (-37.09, 17.61) |
|  | Hospital | $251.79 (1,197.58) | $205.07 (852.00) | $208.83 (1,029.82) | -46.72 (-78.23, -15.20)^b^ | -42.96 (-73.88, -12.04)^b^ | 3.75 (-17.93, 25.43) |
| Other/unknown | | $42.40 (244.21) | $52.39 (1,277.67) | $44.48 (994.47) | 9.99 (-17.34, 37.32) | 2.07 (-12.32, 16.47) | -7.92 (-37.44, 21.61) |
| **Prescriptions** | |  |  |  |  |  |  |
|  | All | $3,177.08 (13,487.24) | $2,922.65 (10,278.22) | $2,849.14 (10,106.30) | -254.43 (-597.52, 88.66) | -327.95 (-644.22, -11.67)^b^ | -73.51 (-336.60, 189.57) |
|  | Obesity-specific | $466.84 (3,047.50) | $391.89 (2,650.45) | $455.23 (3,179.49) | -74.95 (-162.24, 12.35) | -11.61 (-94.91, 71.69) | 63.34 (-6.54, 133.22) |
| Overall services | | $3,884.01 (13,667.04) | $3,502.32 (10,573.89) | $3,414.10 (10,319.24) | -381.69 (-732.06, -31.31)^b^ | -469.91 (-791.45, -148.37)^b^ | -88.22 (-357.82, 181.37) |
|  |  |  |  |  |  |  |  |
| **24 months post-index** | | ESS = 4,482 | ESS =3,941 | ESS =7,397 |  |  |  |
| Inpatient services | | $62.59 (427.96) | $66.81 (699.76) | $48.43 (508.57) | 4.21 (-24.77, 33.20) | -14.16 (-32.84, 4.52) | -18.37 (-46.11, 9.37) |
| Telehealth services | | $9.04 (94.27) | $16.49 (266.18) | $12.91 (171.28) | 7.45 (-3.07, 17.96) | 3.87 (-1.77, 9.50) | -3.58 (-14.51, 7.35) |
| **Outpatient services** | |  |  |  |  |  |  |
|  | All | $1,080.18 (2,060.62) | $999.04 (1,891.52) | $1,005.94 (2,304.98) | -81.14 (-179.23, 16.95) | -74.24 (-168.13, 19.65) | 6.90 (-83.06, 96.86) |
|  | Clinic | $38.86 (308.82) | $41.41 (352.64) | $32.35 (163.54) | 2.55 (-16.64, 21.74) | -6.51 (-15.26, 2.24) | -9.06 (-27.12, 9.00) |
|  | Office | $598.50 (1,399.25) | $561.88 (1,113.84) | $579.94 (1,598.94) | -36.61 (-97.24, 24.01) | -18.56 (-79.30, 42.18) | 18.05 (-37.06, 73.16) |
|  | Hospital | $442.82 (1,250.28) | $395.74 (1,257.77) | $393.65 (1,395.88) | -47.08 (-110.07, 15.92) | -49.17 (-108.65, 10.31) | -2.09 (-58.78, 54.60) |
| Other/unknown | | $80.44 (515.97) | $89.84 (643.09) | $82.88 (367.32) | 9.40 (-14.51, 33.31) | 2.44 (-12.43, 17.31) | -6.96 (-29.52, 15.60) |
| **Prescriptions** | |  |  |  |  |  |  |
|  | All | $7,355.48 (28,977.85) | $6,425.93 (18,267.75) | $6,147.66 (20,163.19) | -929.54 (-2,068.98, 209.89) | -1,207.82 (-2,264.73, -150.91)^b^ | -278.28 (-1,183.54, 626.98) |
|  | Obesity-specific | $1,149.88 (6,678.38) | $875.24 (5,321.86) | $941.44 (5,724.28) | -274.64 (-580.12, 30.85) | -208.44 (-500.04, 83.16) | 66.20 (-185.64, 318.03) |
| Overall services | | $8,587.74 (29,201.78) | $7,598.11 (18,709.51) | $7,297.82 (20,426.95) | -989.63 (-2,148.25, 169.00) | -1,289.92 (-2,358.46, -221.38)^b^ | -300.29 (-1,223.90, 623.31) |

^a^ESS: Effective sample size

^b^Significant at a .05 level

**Table S13**. Prevalence of comorbidities in both Noom Weight cohort and non-Noom cohort

| **Comorbidity** | **Noom users** | **Non-Noom users** |
| --- | --- | --- |
|  | n (%) | n (%) |
| Dyslipidemia | 7,833 (18.2) | 3,130 (21.5) |
| Hypertension | 7,856 (18.2) | 3,568 (24.5) |
| Mental health disorders | 7,473 (17.4) | 2,219 (15.2) |
| Osteoarthritis | 4,506 (10.5) | 1,671 (11.5) |
| Pre-diabetes | 2,635 (6.1) | 896 (6.1) |
| Diabetes mellitus, type 2 | 2,409 (5.6) | 1,372 (9.4) |
| Cardiovascular disease | 1,091 (2.5) | 706 (4.8) |
| Substance abuse (drug or alcohol) | 1,072 (2.5) | 856 (5.9) |
| Joint replacement (lower limb) | 853 (2.0) | 249 (1.7) |
| Hyperglycemia | 808 (1.9) | 340 (2.3) |
| Chronic kidney disease | 497 (1.2) | 283 (1.9) |
| Diabetes mellitus, other | 384 (0.9) | 292 (2.0) |
| Diabetes mellitus, type 1 | 267 (0.6) | 119 (0.8) |
| Tobacco use | 232 (0.5) | 205 (1.4) |
| Rheumatoid arthritis | 63 (0.1) | 46 (0.3) |
| Asymptomatic HIV^a^ | 15 (0.0) | 30 (0.2) |

^a^HIV: Human immunodeficiency virus
